# Lesion location impact on functional recovery of the hemiparetic upper limb

**DOI:** 10.1371/journal.pone.0219738

**Published:** 2019-07-19

**Authors:** Silvi Frenkel-Toledo, Gil Fridberg, Shay Ofir, Gadi Bartur, Justine Lowenthal-Raz, Osnat Granot, Shirley Handelzalts, Nachum Soroker

**Affiliations:** 1 Department of Physical Therapy, Faculty of Health Sciences, Ariel University, Ariel, Israel; 2 Department of Neurological Rehabilitation, Loewenstein Hospital, Raanana, Israel; 3 Sackler Faculty of Medicine, Tel Aviv University, Tel Aviv, Israel; 4 Department of Physical Therapy, Reuth Rehabilitation Hospital, Tel Aviv, Israel; 5 Recanati School for Community Health Professions, Faculty of Health Sciences, Ben-Gurion University of the Negev, Beer-Sheva, Israel; University of Rome, ITALY

## Abstract

The effect of stroke topography on the recovery of hemiparetic upper limb (HUL) function is unclear due to limitations in previous studies–examination of lesion effects only in one point of time, or grouping together patients with left and right hemispheric damage (LHD, RHD), or disregard to different lesion impact on proximal and distal operations. Here we used voxel-based lesion symptom mapping (VLSM) to investigate the impact of stroke topography on HUL function taking into consideration the effects of (a) assessment time (subacute, chronic phases), (b) side of damaged hemisphere (left, right), (c) HUL part (proximal, distal). HUL function was examined in 3 groups of patients—Subacute (n = 130), Chronic (n = 66), and Delta (n = 49; patients examined both in the subacute and chronic phases)–using the proximal and distal sub-divisions of the Fugl-Meyer (FM) and the Box and Blocks (B&B) tests. HUL function following LHD tended to be affected in the subacute phase mainly by damage to white matter tracts, the putamen and the insula. In the chronic phase, a similar pattern was shown for B&B performance, whereas FM performance was affected by damage only to the white matter tracts. HUL function following RHD was affected in both phases, mainly by damage to the basal ganglia, white matter tracts and the insula, along with a restricted effect of damage to other cortical structures. In the chronic phase HUL function following RHD was affected also by damage to the thalamus. In the small Delta groups the following trends were found: In LHD patients, delayed motor recovery, captured by the B&B test, was affected by damage to the sensory-motor cortex, white matter association fibers and parts of the perisilvian cortex. In the RHD patients of the Delta group, delayed motor recovery was affected by damage to white matter projection fibers. Proximal and distal HUL functions examined in LHD patients (both in the subacute and chronic phases) tended to be affected by similar structures—mainly white matter projection tracts. In RHD patients, a distinction between proximal and distal HUL functions was found in the subacute but not in the chronic phase, with proximal and distal HUL functions affected by similar subcortical and cortical structures, except for an additional impact of damage to the superior temporal cortex and the retro-lenticular internal capsule only on proximal HUL function. The current study suggests the existence of important differences between the functional neuroanatomy underlying motor recovery following left and right hemisphere damage. A trend for different lesion effects was shown for residual proximal and distal HUL motor control. The study corroborates earlier findings showing an effect of the time after stroke onset (subacute, chronic) on the results of VLSM analyses. Further studies with larger sample size are required for the validation of these results.

## Introduction

Stroke is the leading cause of adult acquired motor disability [1]. Up to 85% of stroke survivors encounter an initial upper limb (UL) motor deficit [2,3]. Long-term sequelae are substantial—55% to 75% endure UL motor deficits 6 months post stroke [4] and up to 50% encounter UL function problems 4 years post stroke [5]. Current rehabilitation methods usually fail to improve HUL function following a severe initial impairment, and consequently, independence in activities of daily living and quality of life remain reduced for most patients with severe hemiparesis [6].

Recovery after stroke is attributed to plastic reorganization in the central nervous system. Reorganization commonly refers to recruitment of areas previously not (or less) engaged in a given task, in order to substitute for directly lesioned or disconnected areas [7,8]. The functional reorganization of the motor system after stroke is constrained most obviously by the exact location and extent of the anatomical damage [9,10]. Previous studies in stroke patients evaluated the effects of lesion characteristics on general functioning of the survivors and on functions of the HUL. Brain regions where damage highly influences general motor function include the corona radiate, internal capsule, insula, superior longitudinal fasciculus, uncinate fasciculus, postcentral gyrus, putamen and operculum [11,12]. Miyai and colleagues [13] found that hemiparesis following cortical damage is generally more likely to improve compared to hemiparesis following subcortical damage. Fries and colleagues [14] found that damage jointly involving the internal capsule, caudate and putamen is associated with better motor recovery compared to damage involving the posterior limb of the internal capsule (PLIC) in combination with the lateral thalamus. In addition, the corticospinal integrity was found to correlate with and predict the motor ability of the HUL in chronic stroke patients [15–18].

Studies that investigate the effect of lesion location on HUL function are usually limited to behavioral testing in one point in time. During the acute phase, the probability of HUL recovery was found to decrease progressively with lesion location as follows: cortex—corona radiate—PLIC [19]. In the sub-acute phase, damage to subcortical structures showed higher association with poor motor performance of the HUL [20]. In the chronic phase, a more posterior lesion within the PLIC was found to correlate with severe residual HUL impairments and disability [21]. Thus, the impact of the exact location of stroke-related brain damage on HUL motor function might differ when testing is done at different times post stroke onset, in accord with the amount of adaptive plasticity taking place up to the time of testing [22].

Differences in recovery of the distal and proximal HUL motor functions are likely to relate to the greater precision and fractionation of muscular activity needed for distal (grasp, pinch) operations. This is reflected in the larger extent of cortical representation of the distal UL (hand, fingers) relative to the proximal UL (shoulder, elbow) in the primary motor cortex [23], and in distinct cortical activation patterns during distal and proximal UL movements [24]. In addition, while the cortical output for execution of distal UL movements is channeled mainly via the lateral cortico-spinal tract, proximal and trunk movements are controlled to a large extent by the anterior (ventral, non-crossing) cortico-spinal tract and the brainstem descending tracts that maintain a more widespread bilateral innervation at the spinal level [23]. Indeed, the clinical manifestation of impaired motor ability following stroke might differ significantly in the proximal and distal parts of the HUL, and the responsiveness of the two segments of the HUL to rehabilitation interventions may also differ [25]. Most studies that assessed the effect of lesion characteristics on HUL motor function [19–21] did not attempt to differentiate the effect on proximal UL function (postural setting, reaching) and distal UL function (grasp). Schiemanck and colleagues [26] studied lesion effects on distal HUL function and showed that at one-year post onset, lesion to the internal capsule was associated with a significantly lower probability of return of isolated hand/fingers movements as compared to lesions of the cerebral cortex, adjacent subcortical regions and the corona radiate.

The significance of right/left hemispheric differences in the organization of brain functions is well acknowledged in lesion studies addressing cognitive or linguistic functions [27–29]. In contrast, lesion studies investigating the effects of stroke location on motor ability often address the right and left hemispheres (RH, LH) as two parallel and analogous systems. Some studies analyzed lesion-symptom relationship in right and left hemispheric groups separately and thereafter collapsed the results into a single group [14,19–21]. In some studies, lesion imaging data was flipped to one side, in order to group subjects together prior to analysis, to increase the statistical power [11,30,31]. It has been suggested though, that such flipping might obscure important differences, and indeed separate analyses of hemispheric groups yielded dis-similar patterns [12]. Thus, in the LH, white matter lesions (corona radiata, internal and external capsules, superior longitudinal fasciculus, uncinate fasciculus) as well as damage to the postcentral gyrus, putamen, and operculum, were found to be associated with poorer performance at the subacute phase, whereas in the RH, lesions to the insula, operculum, and putamen were more likely to be associated with severe long-term disability [12]. Cortical reorganization after stroke was also shown to be affected by lesion side [32], a finding of relevance for evaluation of lesion-symptom relationships following long-term recovery in the chronic stage. Taking all the above into consideration, we chose to analyze and discuss right and left sided lesions separately.

The goal of post-stroke HUL rehabilitation is to trigger and facilitate processes of adaptive neural plasticity, in order to lessen the level of sensory-motor impairment and thereby decrease activity limitation and restrictions to participation that stem from the HUL impaired functioning [33,34]. Widespread clinical application of experimental adjuvant therapies, such as trans-cranial electrical or magnetic stimulation (tDCS [35], TMS [36]) or mirror visual feedback [37], is attenuated by lack of sufficient understanding of the reasons for the large inter-personal variance shown in the responsiveness of stroke patients to such interventions. This variance is likely to stem, at least in part, from differences in lesion location and extent [38]. Research aimed to uncover the ways motor recovery is affected by different lesion patterns is extremely relevant for the emerging field of plasticity modulation in neuro-rehabilitation.

The aim of the current study was to investigate the relationship between lesion characteristics and HUL function following stroke. Previous lesion studies usually (a) addressed general HUL function without differentiation between reach and grasp (proximal, distal) operations; (b) were limited to a specific time interval, that is—correlated lesion data with function measured either early after onset or in the chronic stage; (c) did not separate between right and left hemispheric damage. In the current study, we examined the effects of lesion characteristics on motor functioning of the HUL, as revealed in different points in time after onset (subacute and chronic phases, and the dynamics in the time interval between these phases), while differentiating between proximal and distal HUL functioning. Lesion effects on HUL function were analyzed separately in patients with right and left hemispheric stroke.

We conjectured that HUL function in the subacute phase is constrained mainly by damage to cortical and subcortical structures directly involved in normal motor execution, namely, the UL part of the homunculus in the primary motor cortex (pre-central gyrus) and the corticospinal tract in its passage through the corona radiata and the PLIC [23]. We also assumed that HUL function in the subacute phase, as well as later in the chronic phase, is constrained in addition by (1) damage to peri-lesional cortical regions that typically take over motor functions after damage to motor cortices (i.e., functional re-mapping processes underlying recovery) [39], and (2) by damage to structures that play a role in procedural learning and the acquisition of new motor routines (basal ganglia, pre-motor cortical regions). In as much as the operation of processes of adaptive neuroplasticity are delayed (since neuroplasticity processes occur also in the chronic phase [40,41]), the impact of damage to the brain structures involved in such processes is expected to be revealed in VLSM analyses conducted in the chronic phase rather than in the subacute phase, and in the delta between the performance early and late after stroke onset. With respect to proximal vs. distal UL motor function, we expected distal function to be more prone to remain compromised (following both cortical and subcortical damage) than proximal function, due to the bilateral control of proximal UL motor functioning (both contralateral and ipsilateral tracts are involved with proximal motor control) [23].

## Method

### Participants

Three groups of first-event stroke patients hospitalized in the Loewenstein Rehabilitation Hospital, Ra’anana, Israel, were recruited for the study. 130 patients were tested shortly after stroke onset ("Subacute" group–average time after onset [TAO] less than 1.3 months, ranging from 0.5 to 3 months), 66 patients were tested in the chronic phase of the disease ("Chronic" group–average TAO more than 2 years, ranging from 10.2 to 68.5 months), and 49 patients were tested during both the subacute and the chronic phases ("Delta" group). Patients were considered to be in the subacute phase if they were 3 months or less after stroke onset at the time of testing [42], and in the chronic phase if they were at least 6 months after stroke onset [43]. Patients were included in the Chronic and Delta groups only if they did not have a subsequent stroke. Patients were included in the study if they did not suffer from previous psychiatric or neurological disorders and their language and cognitive status enabled comprehension of the task requirements. The study was approved by the Ethics Review Board of the Loewenstein Hospital. Patients who had the capacity to provide informed consent, which was determined based on an individual’s ability to respond to questions and engage in conversation by a medical rehabilitation doctor and a physical therapist, were suggested to participate in the study after receiving an explanation about the study. They were told that they are not obliged to participate and that they could quit whenever they want. All patients signed an informed consent prior to recruitment for the study. Consent for publication of raw data was not obtained but the dataset is fully anonymous in a manner that can easily be verified by any user of the dataset. Publication of the dataset clearly and obviously presents minimal risk to confidentiality of study participants. It was not possible to obtain from participants consent for publication of the fully anonymous raw-data, as an explicit statement on raw data publication is not part of the detailed standard form used for obtaining informed consent from participants upon recruitment for studies at the Loewenstein Rehabilitation Hospital (LRH), and it is impossible to obtain consent on this specific statement in retrospect. However, the consent was given based on explicit written declaration of the researchers not to publish any information that could unravel the identity of any participant, and this was strictly kept. All measures have been taken to remove from the deposited raw data information that could lead to identification of specific participants, in consultation with the LRH ethics review board.

Patients’ demographic and clinical data are described in Table 1. Thirteen patients with RHD and 2 patients with LHD from the Subacute group, and 6 patients with RHD and 2 patients with LHD from the Chronic group had neglect (according to the Behavioral Inattention Test; [44,45]). 33, 24 and 18 patients with LHD from the Subacute, Chronic and Delta groups, respectively, had aphasia (according to the Israeli Loewenstein Aphasia Test; [46]). Individual data are displayed in S1 Table.

**Table 1:** Demographic and clinical characteristics of participants.

	Subacute Group			Chronic Group			Delta Group		
Lesion side	Right (n = 65)	Left (n = 65)	p. value	Right (n = 32)	Left (n = 34)	p. value	Right (n = 24)	Left (n = 25)	p. value
**Gender (M/F)**	43/22	45/18	0.447^a^	26/6	26/8	0.635^a^	17/7	21/4	0.269^a^
**Age: Mean (SD)**	60.94 (9.92)	60.17 (11.58)	0.204^c^	62.97 (9.76)	60.13 (10.40)	0.132^c^	60.54 (9.97)	58.16 (10.84)	0.427^b^
**Dominance (R/L/A)**	60/3/2	62/3/0	0.362^a^	29/2/1	32/2/0	0.580^a^	22/1/1	25/0/0	0.338^a^
**Lesion type (I/H/I>H)**	47/16/2	47/17/1	0.834^a^	20/9/3	21/13/0	0.158^a^	16/6/2	13/12/0	0.117^a^
**TAO months**	1.34 (0.64)	1.44 (0.59)	0.221^c^	29.31 (14.33)	28.40 (14.46)	0.807^c^	27.64 (15.15)	27.48 (15.07)	0.912^c^
**Lesion volume: cc**	25.48 (36.35)	20.12 (23.30)	0.497^c^	30.90 (41.88)	23.78 (26.86)	0.868^c^	29.40 (42.26)	22.42 (22.76)	0.424^c^
**FM A: X/30 (SD)**	16.92 (11.00)	17.38 (9.79)	0.884^c^	20.84 (12.17)	21.91 (8.30)	0.449^c^	2.38 (4.19)	5.92 (7.64)	0.106 ^c^
**FM B+C: X/24 (SD)**	12.83 (9.65)	13.11 (9.05)	0.973^c^	15.91 (10.11)	16.29 (8.20)	0.687^c^	1.71 (4.17)	3.40 (6.73)	0.291^c^
**FM Total: X/66 (SD)**	36.55 (22.53)	36.71 (21.19)	0.985^c^	44.31 (24.57)	45.79 (17.85)	0.492^c^	4.04 (9.69)	9.76 (14.91)	0.186^c^
B&B*: Mean (SD)	19.95 (20.19)	21.13 (19.13)	0.703^c^	28.59 (22.76)	30.45 (23.32)	0.772^c^	3.4 (12.87)	8.88 (9.61)	0.264^b^
**FM Sensation: X/12(SD)**	8.77 (3.90)	9.90 (3.31)	0.129^c^	8.93 (3.53)	9.41 (3.97)	0.394^c^	1.46 (4.31)	1.13 (2.10)	0.686^c^
**VFD (no/extinction/yes)**	56/6/3	59/3/3	0.583^a^	26/5/1	33/1/0	0.109^a^	20/3/1	24/1/0	0.310^a^

Dominance R = Right, L = Left, A = Ambidextrous; Lesion type I = Ischemic, H = Hemorrhagic, I>H = Ischemic with hemorrhagic transformation; TAO = Time after stroke onset–mean (SD); FM = Fugl-Meyer, B&B = Box and Blocks; VFD—visual field defect

* Number of participants in the B&B test: in the subacute phase—n = 38 (right) and n = 48 (left), in the chronic phase—n = 29 (right) and n = 29 (left), in the delta–n = 10 (right) and n = 16 (left); Number of participants in the FM sensation test: in the subacute phase—n = 43 (right) and n = 41 (left), in the chronic phase–n = 28 (right) and n = 27 (left), in the delta–n = 10 (right) and n = 16 (left); for the Delta group—TAO, FM, B&B and sensation data are mean and SD values of the difference (delta) in scores from the subacute to the chronic phase, and values of Age and VFD are of the subacute phase. a = Chi-Square test, b = t test, c = Mann Whitney test.

### Clinical assessment

The standardized Fugl-Meyer (FM) [47,48] and Box & Blocks (B&B) [49] tests were used for evaluation of HUL motor function. The total score (66) of the FM test and its sub-scores of proximal and distal parts were used as previously employed [25–26,50]. The sub-scores for the proximal part (FM-A) include items related to movements of the shoulder, elbow and forearm (FM part A, without reflex assessment, 15 items). The sub-scores of the distal part (FM-B+C) include items related to hand (part B, 5 items) and fingers (part C, 7 items). The scale has proven to be sensitive, reliable and valid [51,52]. The B&B is another widely used, reliable and valid measure of HUL functioning, where the score is based on the number of blocks (size: 2.54 cm^3^) that can be transported from one compartment of a box to another compartment within 1 minute. It is a measure of gross manual dexterity [49].

Subacute and Chronic groups were assessed 1.4 ± 0.6 months (range: 0.5–3.0) and 28.8 ± 14.3 months (range: 10–68) after the onset of stroke, respectively. Members of the Delta group (i.e., patients for whom data is available from both testing periods) had their second testing in the chronic phase at 28.9 ± 15.1 months (range: 22.6–66.1) after stroke onset. The delta score for each patient was defined as the change between the Fugl-Meyer (FM) score obtained during the chronic and the subacute phases (FM delta = FM chronic—FM subacute; for a similar approach see [53,54]).

### Imaging

Follow-up CT scans dated on average 45, 47 and 46 days post stroke onset for the Subacute, Chronic and Delta groups, respectively, were carefully examined by a physician experienced in analysis of neuro-imaging data (NS) in order to ensure that lesion boundaries were clear and traceable and that the CT presents a stable pattern of tissue damage without a mass effect from residual edema.

#### Lesion analysis

**Fig 1:**
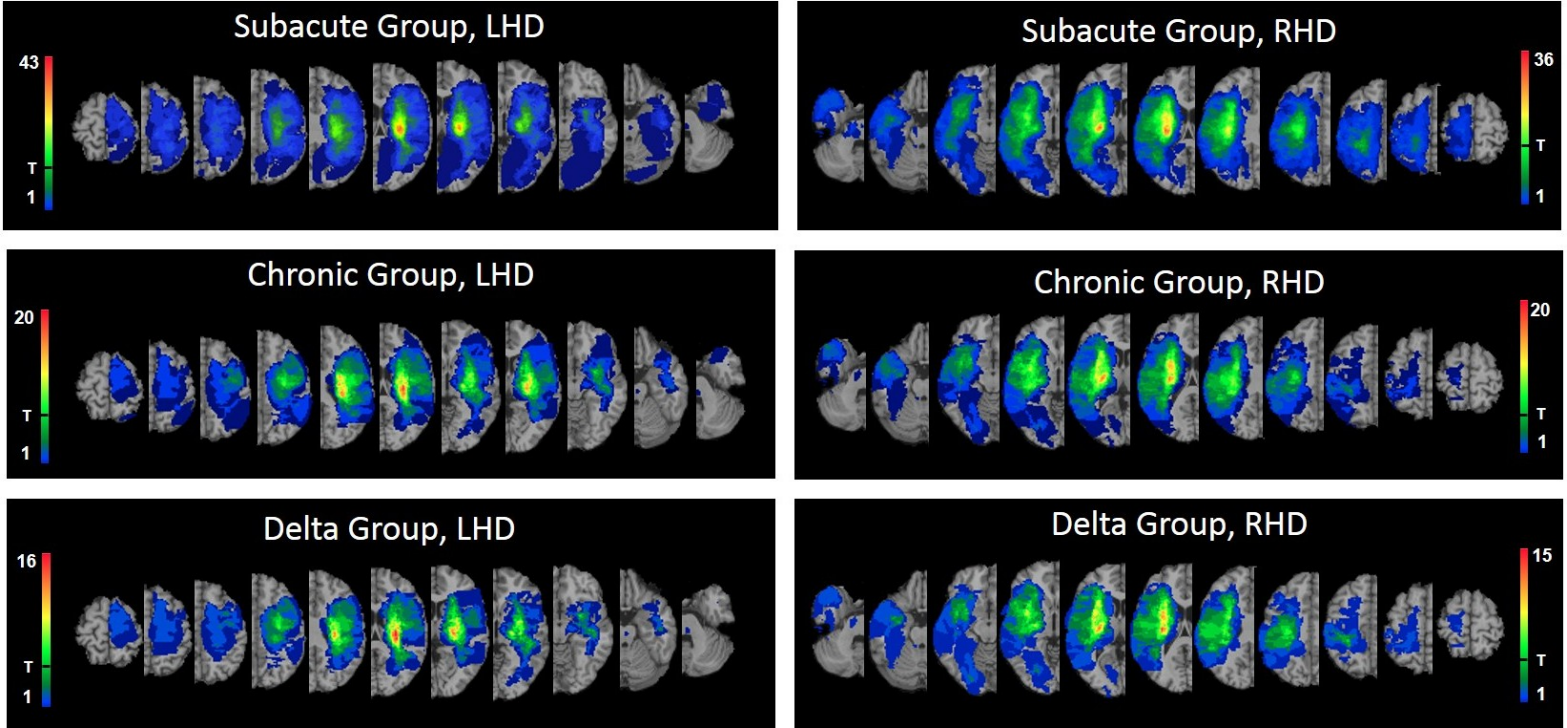
Lesion overlay maps of LHD and RHD patients of the Subacute (n = 65, n = 65; respectively), Chronic (n = 34, n = 32; respectively) and Delta (n = 25, n = 24; respectively) groups. T = threshold for inclusion in the VLSM analysis: at least 20% of the subjects had to have damage to a particular voxel for it to be included in the analysis. Regions analyzed in each of the 6 figures are those coded by colors above the T mark. Representative normalized slices (out of 90 normalized slices employed) are displayed in radiological convention (right hemisphere on left side and vice versa), with warmer colors indicating greater lesion overlap (units: number of patients with lesion in this region).

Lesion analyses were performed with the Analysis of Brain Lesions (ABLe) module implemented in MEDx software (Medical-Numerics, Sterling, VA, USA). Lesion delineation was made manually on the digitized CTs. ABLe characterizes brain lesions in MRI and CT scans of the adult human brain by spatially normalizing the lesioned brain into Talairach space using the Montreal Neurological Institute (MNI) template. It reports tissue damage in the normalized brain using an interface to the Talairach Daemon (San Antonio, Texas) [55], Automated Anatomical Labeling (AAL) atlas [56,57], Volume Occupancy Talairach Labels (VOTL) atlas [55,56] or the White Matter Atlas [58]. Quantification of the amount of tissue damage within each structure/region of the atlas was obtained as described earlier [59]. In the current study, tissue damage in the normalized brain was reported using the interface to the AAL atlas and white matter atlas. Registration accuracy of the scans to the MNI template across all subjects ranged from 89.1% to 95.9% (Subacute group: 94.1 ± 1.2, 94.2 ± 1.1 in LHD and RHD subjects, respectively; Chronic group: 94.1 ± 1.4, 94.5 ± 0.8 in LHD and RHD subjects, respectively; Delta group: 94.1 ± 1.4, 94.4 ± 0.9 in LHD and RHD subjects, respectively). Individual lesion data are displayed in S1 Fig. Overlay lesion maps (stroke lesion distribution) of LHD and RHD patients in the 3 groups (Subacute, Chronic and Delta) are shown in Fig 1.

#### Voxel-based Lesion-Symptom Mapping (VLSM)

Voxel-based Lesion Symptom Mapping (VLSM; [60]) was used to identify voxels of the normalized brain where damage has a significant impact on the FM and B&B scores. Voxel-by-voxel analysis calculates the statistical significance of the difference (using t-tests) in performance between participants with damage in a given voxel and participants who are not damaged in that voxel. At least 20% of the subjects had to have damage to a particular voxel, for it to be included in the analyses. By setting this threshold we aimed to increase the statistical power by reducing the number of comparisons in need of correction (in the analysis of the B&B test in the RHD Delta group, where the number of subjects was small (n = 10), a voxel was analyzed if at least 3 subjects had damage to that particular voxel [59]). At least 10 adjacent voxels had to show a statistically significant impact on performance for a cluster to be reported [61]. To correct for multiple comparisons, voxels with values exceeding a false discovery rate (FDR) / permutation threshold of p < .05 were considered significant [62]. Due to insufficient statistical power in part of the analyses, we also report anatomical regions containing clusters of at least 10 voxels, where patients affected in these voxels showed disadvantage relative to patients who were not affected in these voxels, using a lenient criterion of p < .01, which did not survive FDR correction for multiple comparisons (for a similar approach see references [30, 63–65]). This information is provided under the assumption that in such cases VLSM points to possible trends. The maximum z-score is reported for each cluster of contiguous above-threshold voxels. Since there may be multiple voxels with this maximum z-score in the cluster, we report the coordinate of the voxel that is most superior, posterior and left in its location within the cluster (the centroid of the cluster is not reported as it may not have the highest z-score value and it may not be an above-threshold voxel). The Automated Anatomical Labeling atlas (AAL) atlas for gray matter and the white Matter Atlas [55–58] were used to identify the location of the significant clusters.

In order to rule out the possibility that the results were influenced differently in the RHD and LHD groups by the demographic and clinical characteristics, the gender, age, dominance, lesion type, time after stroke onset, lesion volume, FM A, FM B+C, FM T, B&B, FM sensation, and the prevalence of visual field defects were compared between groups, in each phase, using t-tests or Mann-Whitney tests or Chi-square tests as required (normal group distribution of continuous data was assessed using Kolmogorov–Smirnov tests). A comparison was made between the RHD and LHD groups with respect to (1) the proportion of subjects affected in each region of the AAL and WM atlases, and (2) the extent of damage in each region, using Chi-square/Fisher’s exact tests, and Mann Whitney tests, respectively. In addition, correlations between total lesion volume and the clinical test scores were calculated in both groups, using Spearman-rho. FDR was used to correct for multiple comparisons. All the tests were done using SPSS (version 25.0) with significance levels of p<0.05.

## Results

Demographic and clinical characteristics of RHD and LHD patients were essentially similar, both in the subacute and in the chronic phases (Table 1). In the subacute phase, the proportion of subjects having a lesion (yes/no) in each of the regions of the AAL and WM atlases was similar. In the chronic phase and also among the ‘Delta’ group, there was a larger proportion of LHD patients with damage to the caudate nucleus (74% vs. 44% in the chronic phase and 76% vs. 42% in the ‘Delta’ groups, FDR corrected p value < 0.05; the proportions of patients affected in all the other regions were similar in LHD and RHD patients). The extent of damage was greater in RHD compared to LHD patients in the orbital part of the superior and middle frontal gyri, the anterior cingulum, the parahippocampal gyrus and the middle cerebellar peduncle. In contrast, LHD patients had bigger lesions compared to RHD patients in the posterior and retro-lenticular parts of the internal capsule (PLIC, RLIC)–this difference was evident in our sample only in the subacute phase. In the RHD group, total hemispheric volume loss correlated significantly (negative correlation) with the following clinical test scores: FM A, FM B+C, FM T tested in the subacute phase (Spearman-rho values -0.462, -0.403 and -0.458, respectively); FM A, FM B+C and B&B tested in the chronic phase (Spearman-rho values -0.444, -0.453 and -0.556, respectively). All other correlations between total lesion volume and test results were not significant.

**Fig 2:**
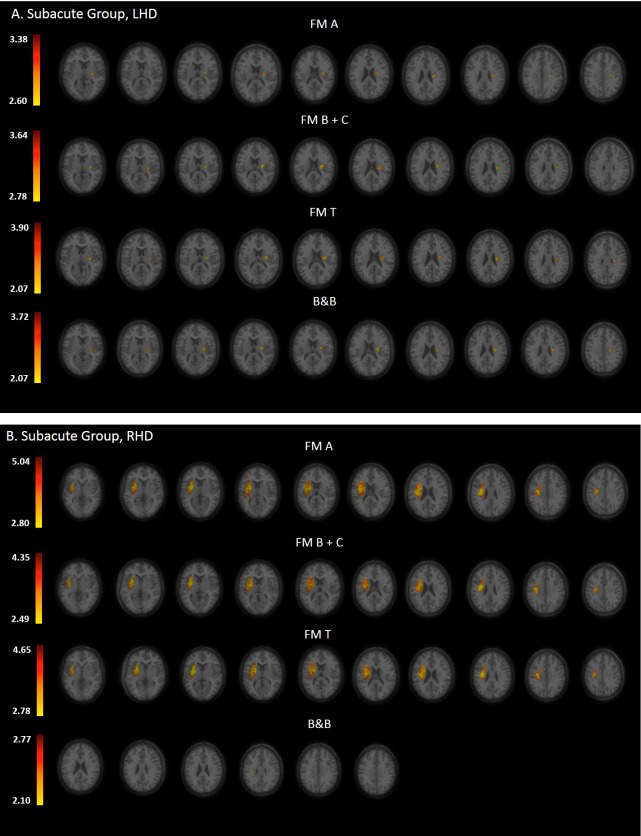
**VLSM analysis depicting areas of damage that were associated with lower FM A, B+C, T and B&B scores in the LHD (A) and RHD (B) groups during the subacute phase.** Colored regions denote voxels where damage exerted a significant impact on behavioral scores, based on a lenient criterion of z score = 2.00 (p = 0.01) or above in the LHD group (all clinical assessment scales) and B&B of the RHD group. The colored regions in FM A, B+C, T of the RHD group survived FDR correction for multiple comparisons. Voxels were analyzed only if they were damaged in at least 20% of the sampled stroke patients. Significant voxels are reported only if they are part of clusters comprising at least 10 voxels. Regions in red correspond to higher z scores.

**Fig 3:**
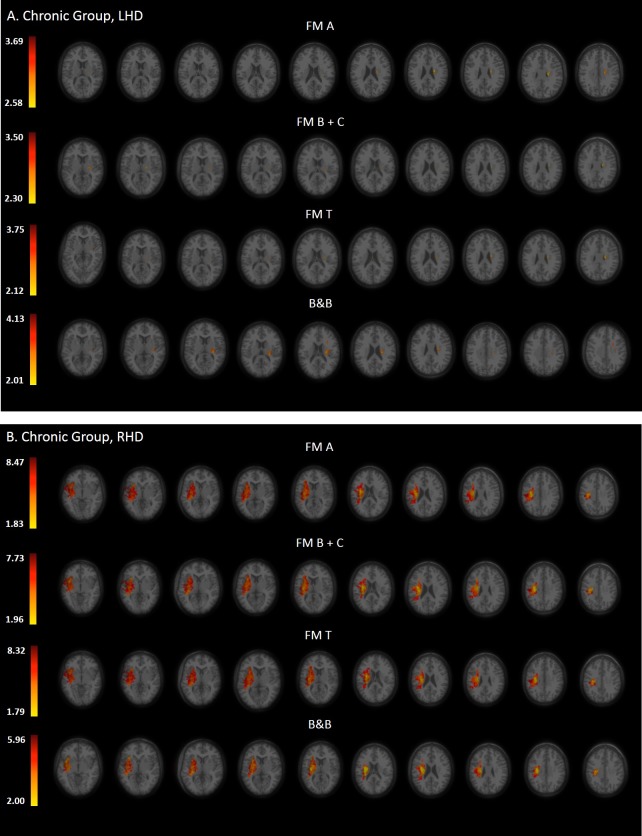
**VLSM analysis depicting areas of damage that were associated with lower FM A, B+C, T and B&B scores in the LHD (A) and RHD (B) groups during the chronic phase.** Colored regions denote voxels where damage exerted a significant impact on behavioral scores, based on a lenient criterion of z score = 2.00 (p = 0.01) or above in the LHD group (all clinical assessment scales), and FDR correction for multiple comparisons in the RHD group (all clinical assessment scales). Voxels were analyzed only if they were damaged in at least 20% of the sampled stroke patients. Significant voxels are reported only if they are part of clusters comprising at least 10 voxels. Regions in red correspond to higher z scores.

**Fig 4:**
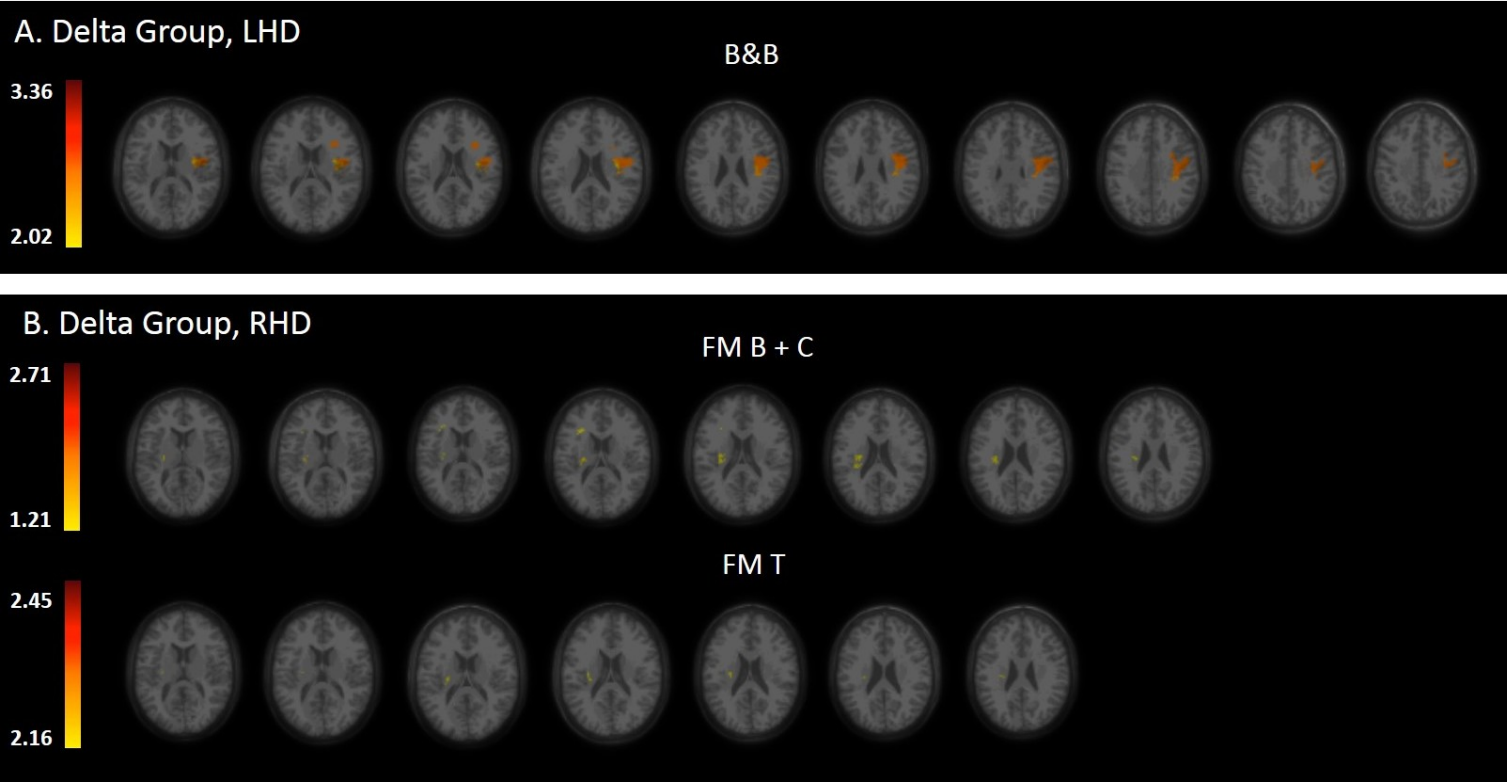
**VLSM analysis depicting areas of damage that were associated with lower B&B gain in the LHD group (A) and lower FM B+C and FM T gains in the RHD group (B) in the time interval between the subacute- and chronic-phase examinations**. Colored regions denote voxels where damage exerted a significant impact on behavioral scores, based on a lenient criterion of z score = 2.00 (p = 0.01) or above in the LHD and RHD groups. Voxels were analyzed only if they were damaged in at least 20% of the sampled stroke patients. Significant voxels are reported only if they are part of clusters comprising at least 10 voxels. Regions in red correspond to higher z scores.

Statistical maps derived from the VLSM analyses, presenting the z scores of voxels whose damage was found to exert a significant impact on the tested motor behaviors of the HUL, are shown in Figs 2, 3 and 4 (Subacute, Chronic and Delta groups, respectively).

Tables 2–7 summarize the following lesion characteristics: (a) cluster location in coordinates of the normalized [MNI, Montreal Neurological Institute] brain; (b) z-values derived from the VLSM analyses; (c) number of significant voxels [voxels whose damage exerted a significant impact on the HUL tested motor behavior] within the boundaries of involved anatomical regions; and (d) the percentage of the volume of the involved anatomical regions which is occupied by significant voxels. Only structures containing 10 or more significant voxels are shown (significant means p < .05 following FDR correction for multiple comparisons, or a more lenient criterion of z-value 2.0 or above which corresponds roughly to the lower z-value of voxels passing the FDR criterion. Regions that passed permutation correction are mentioned as well in the tables and described in detail in supplementary S2 and S3 Tables). The structures affecting proximal upper-limb (UL) function (FM A), distal UL function (FM B+C) and the B&B test are listed in descending order of significant voxels per structure. Note that significant voxels in a structure could belong to one or more distinct clusters.

**Table 2:** VLSM results in LHD patients (n = 65) at the subacute phase.

Test	Structure	Z-value	X	Y	Z	Voxels	% area
**FM A**	SCR	3.38	-26	-16	30	87	9.42
	PLIC	3.22	-26	-12	18	28	5.87
	EC	3.15	-30	-16	10	20	4.44
	SLF	2.94	-32	-12	26	13	1.60
	Putamen	2.92	-28	-12	10	11	1.09
**FM B+C**	SCR	3.64	-26	-16	22	124	13.42
	PLIC	3.59	-26	-12	18	32	6.71
	EC	3.37	-28	-10	14	32	7.11
	SLF	3.00	-32	-10	22	23	2.82
	Insula	3.20	-32	-12	20	22	1.18
	Putamen	3.10	-28	-12	10	11	1.09
**FM T**	SCR	3.90	-26	-16	30	147	15.91
	PLIC	3.63	-26	-12	18	40	8.39
	EC	3.59	-28	-10	14	30	6.67
	Putamen	3.33	-28	-12	10	16	1.59
	SLF	2.52	-32	-8	22	10	1.23
**B&B**	SCR	3.72	-28	-14	28	125	13.53
	Insula	3.28	-32	-14	20	36	1.94
	EC	3.25	-28	-10	16	35	7.78
	PLIC	3.62	-24	-10	16	33	6.92
	SLF	3.02	-32	-14	26	14	1.72

The results shown did not survive the FDR correction for multiple comparisons but passed the more lenient criterion of z score = 2.00 or above (the z scores required to pass the correction for multiple comparisons by permutation testing for FM A, FM B+C, FM T and B&B correspond to 3.90, 3.90, 3.87 and 3.58, respectively). EC = external capsule; PLIC = posterior limb of internal capsule; SCR = superior corona radiata; SLF = superior longitudinal fasciculus. Voxel numbers in each test are listed from highest to lowest. For B&B—n = 48.

**Table 3:** VLSM results in RHD patients (n = 65) at the subacute phase.

Test	Structure	Z-value	X	Y	Z	Voxels	% area
FM A*	Putamen^	4.52	26	-2	6	540	50.75
	SCR^	5.04	30	-10	28	536	58.26
	Insula^	4.84	38	0	10	399	22.54
	EC^	4.49	34	-2	8	382	81.97
	SLF^	4.89	34	-4	22	344	41.70
	ACR	3.92	26	20	12	131	15.30
	ALIC^	4.62	18	-2	18	103	25.31
	R. Operculum	4.39	38	-4	16	84	6.31
	PLIC	4.11	24	-4	16	84	16.77
	Caudate^	4.76	20	-4	20	62	6.24
	SFO^	4.76	20	-4	20	44	74.58
	PCR	3.71	26	-22	26	41	9.07
	IFO	3.28	34	8	-8	24	9.13
	IFG-po	3.54	38	14	14	18	1.29
	Pallidum	3.90	24	-4	6	16	5.71
	Heschl gyrus	2.98	34	-24	16	10	4.02
	RLIC	2.80	30	-20	8	10	3.16
FM B+C*	Putamen^	4.21	26	-2	6	531	49.91
	SCR^	4.35	28	-12	28	486	52.83
	Insula	4.14	38	0	10	366	20.68
	EC	3.86	30	12	8	362	77.68
	SLF	4.18	34	-2	22	320	38.79
	ACR	3.54	26	20	12	124	14.49
	ALIC	3.74	18	-2	18	98	24.08
	R. Operculum	3.78	38	-4	16	78	5.86
	Caudate	3.85	20	-4	20	57	5.73
	PLIC	3.66	22	0	12	57	11.38
	SFO	3.89	22	-4	22	42	71.19
	PCR	3.20	30	-22	30	31	6.86
	IFO	2.98	34	8	-8	22	8.37
	Pallidum	3.79	24	0	6	16	5.71
	IFG-po	3.09	38	14	14	10	0.71
FM T*	Putamen^	4.44	26	-2	6	539	50.66
	SCR^	4.65	30	-14	30	526	57.17
	Insula^	4.56	38	0	10	389	21.98
	EC^	4.21	34	-2	8	374	80.26
	SLF^	4.51	34	-2	22	337	40.85
	ACR	3.79	26	20	12	131	15.30
	ALIC^	4.17	18	-2	18	101	24.82
	R. Operculum	4.15	38	-4	16	83	6.24
	PLIC	3.89	22	0	12	80	15.97
	Caudate^	4.29	20	-4	20	59	5.94
	SFO	4.29	20	-4	20	43	72.88
	PCR	3.26	28	-22	26	40	8.85
	IFO	3.17	34	8	-8	24	9.13
	Pallidum	3.93	24	0	6	16	5.71
	IFG-po	3.42	38	-14	14	12	0.86
**B&B**	SCR	2.77	30	-20	28	19	2.07

* FM A, FM B+C and FM T shown results passed FDR correction for multiple comparisons (corresponding in this analysis to z scores of 1.70, 1.74 and 1.71, respectively). B&B did not survive the FDR correction but passed the more lenient criterion of z score = 2.00 or above (the z scores required to pass the correction for multiple comparisons by permutation testing for FM A, FM B+C, FM T and B&B correspond to 3.73, 3.68, 3.65 and 3.28, respectively). ACR, PCR, SCR = anterior, posterior, superior corona radiata; ALIC, PLIC, RLIC = anterior, posterior, retrolenticular limb of internal capsule; EC = external capsule; IFO = inferior fronto-occipital fasciculus; IFG-po = inferior frontal gyrus pars opercularis; R. operculum = rolandic operculum; SFO = superior fronto-occipital fasciculus; SLF = superior longitudinal fasciculus. Voxels`number in each behavior is listed from highest to lowest.

^Regions that passed permutation correction are mentioned as well (their z-values, x, y, z, voxels and % regions according to permutation correction are described in S2 Table). For B&B—n = 38.

**Table 4:** VLSM results in LHD patients (n = 34) at the chronic phase.

Test	Structure	Z-value	X	Y	Z	Voxels	% area
**FM A**	SCR	3.69	-22	-10	28	86	9.31
**FM B+C**	SCR	3.40	-22	-10	28	35	3.79
	PLIC	2.84	-18	-10	12	31	6.50
		3.50	-18	-14	-4	15	3.14
**FM T**	SCR	3.75	-22	-10	28	72	7.79
	PLIC	2.46	-22	-14	14	21	4.40
		3.24	-18	-14	-4	12	2.52
**B&B**	SCR	4.13	-26	-16	22	141	15.26
	PLIC	2.88	-26	-16	14	56	11.74
	Insula	2.86	-34	-10	22	49	2.64
	EC	2.95	-28	-20	16	33	7.33
	Putamen	2.93	-30	-14	8	17	1.68
	R. Operculum	2.94	-40	-16	22	17	1.72
	RLIC	3.09	-28	-24	12	15	4.82
	SLF	2.86	-34	-10	22	11	1.35

The results shown did not survive the FDR correction for multiple comparisons but passed the more lenient criterion of z score = 2.00 or above (the z scores required to pass the correction for multiple comparisons by permutation testing for FM A, FM B+C, FM T and B&B correspond to 4.23, 4.24, 4.25 and 4.03, respectively). EC = external capsule; SCR = posterior, superior corona radiata; SLF = superior longitudinal fasciculus; PLIC, RLIC = posterior, retrolenticular limb of internal capsule; R. operculum = rolandic operculum. Voxel numbers in each test are listed from highest to lowest (note that significant voxels in a structure that belonged to one or more distinct clusters are mentioned from highest to lowest in the same structure). For B&B—n = 27.

**Table 5:** VLSM results in RHD patients (n = 32) at the chronic phase.

Test	Structure	Z-value	X	Y	Z	Voxels	% area
FM A*	Insula^	6.34	34	-12	16	894	50.51
	Putamen^	5.71	28	-10	14	747	70.21
	SLF^	6.95	32	-16	30	623	75.52
	SCR^	8.47	30	-14	30	540	58.70
	EC^	5.71	32	-14	16	445	95.49
	PLIC ^	6.26	26	-12	18	337	67.27
	STG	4.43	40	-30	4	263	8.37
	RLIC^	5.35	36	-26	2	208	65.82
	ALIC	5.51	24	-4	18	138	33.91
	IFO	4.36	36	-6	-8	128	48.67
	ACR	4.46	26	22	6	124	14.49
	PCR^	6.59	26	-22	26	122	26.99
	Pallidum	4.12	26	-10	-4	116	41.43
	Thalamus	4.16	18	-10	12	110	10.41
	R. Operculum	5.21	36	-18	16	101	7.59
	Heschl gyrus	4.83	34	-24	16	100	40.16
	SMG	3.54	38	-24	36	72	3.65
	Postcentral	4.12	36	-24	38	60	1.57
	Caudate	4.78	20	-4	20	57	5.73
	Temp pole sup	1.91	44	4	-14	32	2.39
	SFO	5.65	22	-6	20	27	45.76
	IFG-PO	3.49	38	16	14	22	1.57
	SS	3.46	36	-20	-4	13	4.55
	IFG-PT	3.49	32	24	24	12	0.56
	UNC	2.83	32	8	-14	12	25.53
	Hippocampus	2.83	34	-4	-18	10	1.06
FM B+C*	Insula^	6.43	34	-12	16	917	51.81
	Putamen^	5.56	32	-6	10	766	71.99
	SLF^	6.76	32	-16	30	592	71.76
	SCR^	7.73	26	-16	24	543	59.02
	EC^	5.62	32	-14	16	446	95.71
	PLIC^	5.77	26	-12	18	302	60.28
	STG	4.18	40	-30	4	250	7.96
	RLIC^	5.07	28	-24	14	201	63.61
	ALIC	5.10	24	-4	18	170	41.77
	ACR	4.64	26	28	-2	140	16.36
	Pallidum	3.92	24	-4	6	130	46.43
	IFO	4.64	36	-6	-8	128	48.67
	PCR^	6.11	26	-24	24	120	26.55
	Heschl gyrus	4.76	34	-24	16	108	43.37
	R. Operculum	5.26	36	-18	16	105	7.89
	Thalamus	3.97	18	-10	12	84	7.95
	Caudate	4.58	20	-4	20	83	8.35
	SMG	3.55	38	-24	36	64	3.24
	Postcentral	4.20	36	-24	38	60	1.57
	Temp pole sup	2.01	44	4	-14	32	2.39
	SFO	5.49	22	-6	20	30	50.85
	IFG-PO	3.44	28	16	14	25	1.79
	Hippocampus	3.15	40	-10	-18	14	1.48
	IFG-PT	3.50	32	24	24	12	0.56
	UNC	3.09	32	8	-14	12	25.53
	SS	3.28	36	-20	-4	12	4.20
FM T*	Insula^	6.41	34	-12	16	905	51.13
	Putamen^	5.43	28	-10	14	747	70.21
	SLF^	7.19	32	-16	30	617	74.79
	SCR^	8.32	30	-14	30	541	58.80
	EC^	5.72	32	-14	16	446	95.71
	PLIC^	5.94	26	-12	18	336	67.07
	STG	4.35	40	-30	4	261	8.31
	RLIC^	5.35	36	-26	2	207	65.51
	ALIC	5.28	24	-4	18	138	33.91
	ACR	4.29	26	28	-2	130	15.19
	IFO	4.35	36	-6	-8	128	48.67
	PCR^	6.51	26	-22	26	122	26.99
	Pallidum	4.04	26	-10	-4	116	41.43
	Thalamus	4.03	18	-10	12	110	10.41
	Heschl gyrus	4.94	34	-24	16	110	44.18
	R. operculum	5.25	36	-18	16	102	7.66
	SMG	3.55	38	-24	36	64	3.24
	Caudate	4.60	20	-4	20	60	6.04
	Postcentral	4.14	36	-24	38	60	1.57
	SFO	5.46	22	-6	20	29	49.15
	Temp pole sup	1.89	44	-4	-14	25	1.87
	IFG-po	3.47	38	16	14	21	1.50
	IFG-pt	3.42	32	24	24	12	0.56
	UNC	2.80	32	8	-14	12	25.53
	SS	3.29	36	-20	-4	13	4.55
B&B*	Putamen^	5.96	28	-10	14	722	67.86
	Insula^	5.68	34	-12	16	636	35.93
	SLF^	5.52	32	-16	30	526	63.76
	SCR^	5.96	28	-8	20	478	51.96
	EC^	5.68	32	-14	16	427	91.63
	PLIC^	5.96	28	-12	14	327	65.27
	RLIC	4.25	36	-26	2	205	64.87
	ALIC	4,92	24	-4	18	126	30.96
	IFO	3.78	36	-6	-8	115	43.73
	STG	3.33	40	-30	4	111	3.53
	Pallidum	3.93	26	-10	-4	106	37.86
	Thalamus	3.69	22	-22	12	105	9.93
	PCR^	4.90	26	-22	26	94	20.80
	ACR	3.78	26	28	-2	53	6.19
	Heschl	4.14	34	-24	16	49	19.68
	Caudate	4.32	20	-4	20	47	4.73
	R. Operculum	4.79	36	-18	16	41	3.08
	SFO	5.03	22	-4	20	23	38.98
	Postcentral	3.22	36	-24	38	21	0.55
	Hippocampus	3.12	40	-10	-18	14	1.48
	SS	3.12	38	-28	-4	12	4.20
	UNC	2.79	32	8	-14	12	25.53
	IFG-pt	2.46	32	24	24	10	0.46

* FM A, FM B+C, FM T and B&B shown results passed FDR correction (corresponding in these analyses to z scores of 1.75, 1.75, 1.78 and 1.84, respectively. The z scores required to pass the correction for multiple comparisons by permutation testing correspond to 4.16, 4.24, 4.12 and 3.87, respectively). ACR, PCR; SCR = anterior, posterior corona radiate; ALIC, PLIC, RLIC = anterior, posterior, retrolenticular limb of internal capsule; EC = external capsule; IFO = inferior fronto-occipital fasciculus; IFG-po = inferior frontal gyrus pars opercularis; IFG-pt = inferior frontal gyrus pars triangularis; R. operculum = rolandic operculum; SFO = superior fronto-occipital fasciculus; SLF = superior longitudinal fasciculus; SMG = supramarginal gyrus; SS = sagittal stratum; STG = superior temporal gyrus; UNC = uncinate fasciculus; Temp pole sup = temporopolar region of STG. Voxels`number in each behavior is listed from highest to lowest.

^ = regions that survived also permutation analysis (z-values, x, y, z, voxel numbers, and % of each region affected by significant voxels according to permutation analysis are described in detail in S3 Table). For B&B—n = 29.

**Table 6:** VLSM results in LHD patients (n = 25) of the Delta group.

Test	Structure	Z-value	X	Y	Z	Voxels	% area
**FM A**	VLSM did not yield significant voxels						
**FM B+C**	VLSM did not yield significant voxels						
**FM T**	VLSM did not yield significant voxels						
**B&B**	Precentral	2.02	-52	0	16	262	7.43
	SLF	2.77	-36	-8	24	208	25.52
	Postcentral	2.29	-48	-16	20	159	4.09
	R. Operculum	2.94	-38	-6	20	94	9.49
	Insula	3.36	-36	-10	20	55	2.96
	IFG-po	2.02	-40	2	22	11	1.06

B&B did not survive the FDR correction for multiple comparisons but passed the more lenient criterion of z score = 2.00 or above (the z scores required to pass the correction for multiple comparisons by permutation testing for FM A, FM B+C, FM T and B&B correspond to 2.85, 3.16, 2.84 and 3.20, respectively). IFG-po = inferior frontal gyrus pars opercularis; SLF = superior longitudinal fasciculus; R. Operculum = rolandic operculum. Voxel numbers in each test are listed from highest to lowest. For B&B—n = 16.

**Table 7:** VLSM results in RHD patients (n = 24) of the Delta group.

Test	Structure	Z-value	X	Y	Z	Voxels	% area
**FM A**	VLSM did not yield significant voxels						
FM B+C	SCR	2.71	28	-16	26	44	4.78
	PLIC	2.60	24	-14	18	22	4.39
	PCR	2.50	26	-24	24	10	2.21
**FM T**	SCR	2.43	30	-16	26	18	1.96
**B&B**	VLSM did not yield significant voxels						

FM A, FM B+C and FM T did not survive the FDR correction for multiple comparisons but passed the more lenient criterion of z score = 2.00 or above (the z scores required to pass the correction for multiple comparisons by permutation correction for FM A, FM B+C, FM T and B&B correspond to 3.28, 2.81, 3.27 and 2.01, respectively). SCR, PCR = superior, posterior corona radiate; PLIC = posterior limb of internal capsule. Voxels`number in each behavior is listed from highest to lowest. For B&B—n = 10.

### VLSM conducted in the subacute phase

Tables 2 and 3 show the anatomical structures in the left- and right-hemisphere, respectively, where damage was found to exert a significant impact on HUL function tested in the subacute period.

In the LHD group (Table 2; n = 65), the major impact is attributed to lesions of the white matter pathways, mainly the superior corona radiata (SCR). Both the FM A and FM B+C (proximal and distal UL control, respectively) were affected by lesions to SCR, PLIC, external capsule (EC), superior longitudinal fasciculus (SLF) and the putamen. The distal part was affected also by damage to the insular cortex. It should be noted that these results did not survive the FDR correction for multiple comparisons but passed the more lenient criterion of z score = 2.00 or above.

In the RHD group (Table 3; n = 65) lesion effect differs from the pattern observed in the LHD group. Here the major impact is attributed to lesions of the putamen, white matter pathways and the insular cortex. Both the FM A and FM B+C were affected by lesions to these regions. The proximal part was affected also by damage to Heschl gyrus and RLIC. These results survived FDR correction for multiple comparisons. It should be noted that using permutation analysis, both the FM A and FM B+C (proximal and distal UL control, respectively) were affected by damage to the putamen and the SCR, and the FM A was affected in addition by damage to the insular cortex, other white matter pathways—EC, SLF, anterior limb of internal capsule (ALIC), the superior fronto-occipital fasciculus (SFO)—and also by damage to the caudate nucleus.

### VLSM conducted in the chronic phase

Tables 4 and 5 show the anatomical structures in the left- and right-hemisphere, respectively, where damage was found to exert a significant impact on HUL function tested in the chronic period.

In the LHD group (Table 4; n = 34), the major impact is attributed to lesions of white matter projection pathways—the SCR and PLIC, along with a more restricted impact to damage of the basal ganglia and the insular cortex. Both the FM A and FM B+C (proximal and distal UL control, respectively) were affected by damage to the SCR. The FM B+C was affected also by damage to the PLIC. These results did not survive the FDR correction for multiple comparisons but passed the more lenient criterion of z score = 2.00 or above.

In the RHD group (Table 5; n = 32), lesion effect was found to differ from the pattern observed in the LHD group by showing a prominent impact to damage of the insular cortex, putamen and white matter projection and association pathways (much like the pattern observed for this group in the subacute phase, Table 3), along with an impact to damage of other subcortical and cortical regions. As can be seen in Table 5, both the FM A and FM B+C (proximal and distal UL control, respectively) were affected by damage to a large number of similar subcortical and cortical regions. Contrary to the LHD group, these results survived the FDR correction for multiple comparisons and to a large extent also permutation analysis.

### VLSM conducted on the difference in task performance between the sub-acute and the chronic phase, in patients who could be tested in both phases (Delta group)

Tables 6 and 7 show the anatomical structures in the left- and right-hemispheres, respectively, where damage was found to exert a significant impact on the extent of HUL late recovery (i.e., improvement obtained in the period of time that elapsed between the examination done in the subacute phase and the examination done in the chronic phase).

In the LHD Delta group (Table 6; n = 25), VLSM analysis did not disclose voxel clusters where damage exerted a significant impact on FM scores. The major impact on late amelioration of B&B performance following LHD is attributed to damage of the sensory-motor cortex, and the SLF, along with a restricted impact to damage of the rolandic operculum, the insular cortex and the opercular part of the inferior frontal gyrus (IFG-po). It should be noted that these results did not survive the FDR correction for multiple comparisons but passed the more lenient criterion of z score = 2.00 or above.

In the RHD Delta group (Table 7; n = 24), late recovery of distal motor control (FM B+C) was affected by damage to white matter projection fibers—the SCR, PLIC and PCR. Late increment in FM T was affected by damage to the SCR alone. VLSM analysis did not disclose voxel clusters where damage exerted a significant impact on the FM A and B&B scores. It should be noted that these results did not survive the FDR correction for multiple comparisons but passed the more lenient criterion of z score = 2.00 or above.

## Discussion

The goal of this study was to investigate the effect of lesion location following stroke on motor functioning of the HUL in the subacute and chronic stages, and on the HUL functional recovery (change between the scores obtained during the chronic and the subacute phases). Considering the different anatomical substrates of mechanisms controlling proximal (reach) and distal (grasp) functions of the upper limb [23,26] we investigated lesion effects on these parts separately. Given the differences between the dominant left and the non-dominant right cerebral hemispheres in the functional neuroanatomy of motor control, including known differences in patterns of motor recovery [12,30] we tested lesion effects separately in patients with LHD and RHD. By this we deviated from practice employed in some earlier studies to collapse right and left hemispheric data [11,30,31]. Relative to earlier VLSM studies of lesion effects on motor functions, the sample size in the current study is quite big (130, 66 and 49 first-event stroke patients in the Subacute, Chronic and Delta groups, respectively). However, the decision to analyze lesion effects separately in each hemisphere reduced the numbers and consequently the statistical power of the analyses. For this reason, we report here not only VLSM results that passed corrections for multiple comparisons (FDR / permutations) but also results that passed a more lenient criterion (z-score = 2.0 or higher corresponding to p ≤ 0.01) assuming that these results may point to trends reflecting biological reality. However, since these results did not pass corrections for multiple comparisons, they should be treated with caution and validated in further studies with larger samples.

### Lesion effect on HUL function: Dependence on assessment time

Inference concerning the neural substrate of a given behavior from analysis of stroke lesion data is constrained by the time interval between the occurrence of structural damage (stroke onset) and the time of behavior testing [22]. In the acute stage, task performance is affected not only by direct damage to the neural substrate of the tested behavior. Clinical instability, prevalent at that stage, transient metabolic and physiological instability in regions adjacent to the permanently damaged area, malfunction of anatomically spared components of the motor network due to diaschisis and altered inter-hemispheric balance with inhibition exerted by the contralesional hemisphere–all may affect behavior [41,66–68]. The subacute stage–first weeks and months after stroke onset–is usually the time where functional improvement is most salient, reflecting the changes that occur at this time in the above underlying mechanisms [69]. Resolution of brain edema with improved physiological state in peri-lesional cortex, and plasticity induced by rehabilitation training, are key factors in recovery of function at this stage [67,70]. Lesion effects on behavior tested in the chronic stage differ from the effects observed earlier, due to maturation of re-organization processes in the neural networks that mediate the tested behaviors [22,41]. Thus, the behavioral impact of damage to a given brain structure is more likely to reflect the natural structure-function relationship when task performance is measured shortly after stroke onset and not later in the chronic stage [22]. When task performance is measured at a later stage, especially following successful rehabilitation, deviation from the original structure-function relationship, based on functional re-mapping, is expected [22]. Yet, when damage to a given structure continues to exert a strong impact on task performance, integrity of the damaged structure is more likely to be indispensable for proper functioning of the tested behavior.

In the current study, we did not examine lesion effects in the acute phase. The first FM and B&B testing of the HUL took place in the sub-acute phase (in the mean, about 5 weeks after onset), when the patients were hospitalized for rehabilitation. Therefore, task performance at this stage reflects lesion-location effects, plus the early effects of intensive rehabilitation treatment aimed to facilitate adaptive neuroplasticity and functional recovery. The second FM and B&B testing of the HUL took place in the chronic stage (in the mean, about 30 months after onset). Despite the paucity of long-term treatment, additional improvement in HUL function can be seen (Table 1): both the FM T and B&B improved by 8–9 points in the mean. In the relatively small group of subjects in whom it was possible to conduct the tests both in the sub-acute and in the chronic periods (the Delta group), patients also showed improvement in the time interval between tests (Table 1): the FM T improved by 7 points in the mean, and the B&B improved by 6 points in the mean. Interestingly, the improvement shown in the LHD Delta group was more than twice compared to the improvement shown in the RHD Delta group. However, due to large inter-personal variance, this difference did not reach significance. Past longitudinal studies reported as well on motor improvement that occurred in the subacute and in the chronic phase in some patients [40,69,71,72]. Whereas measuring recovery from the acute phase was out of the scope of this study, others found that the most dramatic recovery of impaired motor functions occurs in the first 30 days after stroke [71].

VLSM analysis of lesion impact on HUL function revealed differences between the areas where damage affected task performance in the subacute phase and those where damage affected performance in the chronic phase. The differences were more salient in the RHD group.

In the LHD group, analysis in the subacute phase revealed a dominant impact of damage to white matter projection tracts (notably the SCR and PLIC), with an additional impact of damage to white matter association fibers (SLF), the putamen and the insular cortex. In the chronic phase, a close pattern was shown for B&B performance, whereas FM performance was affected at that time only by damage to white matter projection tracts (see Tables 2 and 4 for details of VLSM analyses in LHD patients in the subacute and chronic phases).

In the RHD group, VLSM analysis revealed many more structures where damage exerts a significant impact on motor behavior compared to the LHD group. Here the major impact on HUL function was shown by damage to voxel clusters within the basal ganglia, white matter projection and association tracts, the insular cortex and few other perisilvian cortical regions. In the chronic phase RHD patients were affected by lesions to the same structures that emerged in the subacute period, plus the thalamus and additional white matter tracts and (mainly perisilvian) cortical structures (Tables 3 and 5). The finding that more regions (cortical areas, white matter tracts, basal ganglia and thalamus) affect performance in the chronic phase compared to the subacute phase, shown in the RHD group, points to involvement of a large number of network components in relatively late further improvement of residual HUL function after stroke (for a review of connectivity patterns and functional recovery see Grefkes and Fink [73]). Engagement of a large network in the process at this stage blurs the more restricted natural structure-function relationship and explains why the latter is more likely to be revealed in VLSM conducted very shortly after stroke onset [22]. The finding of delayed impact of thalamic damage is in accord with earlier findings showing that damage involving the thalamus may lead to upper extremity weakness [19,74].

Both in RHD and LHD patient groups, damage to white matter tracts exerted a dominant impact on HUL function. This outcome of the VLSM analysis, which was revealed in the subacute as well as in the chronic phase, may show that the effect of neuroplasticity and rehabilitation can partially compensate for damage to cortical regions but is less effective in compensating for damage to the motor pathways, especially to the descending projection tracts. These findings are in line with VLSM-findings from Lo and colleagues [30] who found that the area most correlated with reduced UL function in the chronic phase was the junction of the corona radiata and the corticospinal tract. The ipsilesional corticospinal tract seems to remain a major final common pathway for mediation of cortical control over spinal motor activity relating to HUL movement, and white matter damage remains a dominant factor restricting motor ability throughout the subacute and the chronic phase [20,30]. The impact shown for damage to the SLF, SCR, and the postcentral gyrus is consistent with previous research suggesting that involvement of these structures is specifically related to poor motor performance [12].

Unexpectedly, in view of the central role of neuronal populations within the precentral gyrus in motor control [23], damage to this gyrus was not found here to affect HUL function significantly. This might be explained by the fact that in our subacute groups only 31% and 37% of LHD and RHD patients, respectively, had damage to the precentral gyrus (29% and 31%, respectively in the chronic groups), encompassing usually parts of the gyrus located more ventro-lateral to the ‘hand area’. Yet, impact of damage to adjacent cortical regions, among them the postcentral and supramarginal gyri of the parietal cortex and the posterior inferior frontal cortex (shown here in the RHD group) may point to the importance of these regions in cortical remapping processes that underly HUL motor recovery. Our findings concerning the involvement of cortical structures and white matter pathways beyond the SMC and the corticospinal tract, in HUL function after stroke, are in line with previous evidence for the importance of various anatomical structures in supporting upper limb motor performance [75,76] and recovery [77] after stroke.

The small number of subjects in the LHD (n = 25) and RHD (n = 24) Delta groups may have precluded attainment of significant effects for several functional assessments in the VLSM analysis (even when using the more lenient criterion of z value higher than 2.0 for voxels whose impact on performance did not survive the FDR correction for multiple comparisons). In the LHD Delta group (Table 6), lesions to the precentral and postcentral gyri, adjacent cortical regions and white matter association fibers of the SLF all affected the likelihood of attaining late improvement in B&B performance. It should be noted that damage to precentral and postcentral cortical voxels, shown here to affect late recovery in the LHD Delta group, did not emerge as having a significant impact on HUL performance (neither FM nor B&B scores) in the LHD group VLSM analyses conducted in the subacute and chronic phases. This shows the added value of testing the same patients in different times after stroke onset for the purpose of detecting brain structures that contribute to delayed functional recovery. In the RHD Delta group, however, only damage to projection fibers of the corona radiata and PLIC was found to affect the likelihood of obtaining delayed further improvements in HUL function.

Given the high prevalence of false negative results, especially in VLSM analyses of small samples (due to correction needed for multiple comparisons), it is likely that the above findings reflect only in part the extent of brain tissue and the number of structures that subserve HUL function and its recovery after stroke.

### Effects of lesion location on proximal versus distal HUL motor function

Comparison of the structures where damage had an impact on proximal HUL function (as reflected in the FM A test results) with the structures where damage affected the distal HUL function (as reflected in the FM B+C test results), reveals in the LHD patients tested in the subacute period essentially a similar pattern, showing an impact of damage to white matter tracts and the putamen both on proximal and distal upper limb function and an impact of damage to the insular cortex on the latter only. This may reflect involvement of the insula in mediation of postural setting necessary for distal upper limb control [78,79], or a role of the insular cortex in remapping processes underlying functional amelioration. The implication of a larger number of voxels in distal compared to proximal upper limb control may reflect the greater contribution to the latter from homologous structures in the contra-lesional hemisphere [23]. In the chronic phase, proximal and distal upper limb functioning in LHD patients was affected by damage restricted to white matter projection fibers (SCR in proximal and SCR plus PLIC in distal upper limb function). Proximal-distal distinctions might have been blurred at this stage by the effect of cortical reorganization processes underlying recovery.

In RHD patients, comparison of the structures where damage had an impact on proximal HUL function with the structures where damage affected the distal HUL function, also revealed a similar pattern, except for involvement of the superior temporal cortex and the retrolenticular part of the internal capsule (RLIC) which was restricted to proximal HUL function in the subacute phase, and involvement of the thalamus which was restricted to distal HUL function when the RHD patients were tested in the chronic phase. In the RHD group, lesions to the insula, basal ganglia, white matter projection and association tracts, along with a restricted effect of damage to cortical structures, were dominant in their impact on both proximal and distal HUL function, both in the subacute and in the chronic phases. These VLSM findings are in line with findings of earlier studies that stressed the impact of subcortical damage on long term HUL function [21,26]. Yet, the current findings point to a pattern of structure-function relationship that shows less adherence to the common knowledge concerning proximal-distal differences in functional neuroanatomy (dorsal-ventral representation in SMC, respectively).

In LHD and RHD patients of the Delta group, a comparison of the pattern of damage that impacts the extent of delayed improvement in proximal versus distal HUL function couldn’t be done, as VLSM analyses failed to yield ‘significant” voxels, probably due to the small number of subjects in the LHD (n = 25) and RHD (n = 24) Delta groups.

### Effects of lesion location on HUL function in LHD versus RHD patients

VLSM revealed marked differences in lesion effects between LHD and RHD groups. In the LHD group, both proximal (FM A) and distal (FM B+C) HUL function, as well as the overall measures of HUL function (FM T; B&B), were affected, both in the subacute and in the chronic phases, mainly by damage to white matter tracts, with an additional impact of damage to the basal ganglia and the insular cortex. In the RHD group, HUL function in both phases was affected by the above structures plus a large array of cortical and subcortical structures. It should be noted that in the LHD group the reported VLSM results (Tables 2 and 4) did not pass FDR correction for multiple comparisons and are based on a more lenient criterion of z score = 2.00 or above (p ≤ 0.01). This fact entails the possibility of type-1 (false positive) errors in the results. However, there is reason to believe that the results can be trusted, as damage to the anatomical structures that emerged in the analysis (notably, corona radiata and the posterior limb of the internal capsule) has been reported in earlier studies as having a major impact on motor recovery, in accord with the role of the corticospinal tract that descends through these regions, as the main pathway whereby spinal motor activity is controlled by cerebral processing [11,18,30,80–83]. In contrast, the reported VLSM results in the RHD group did survive FDR correction for multiple comparisons and in part also correction by permutations analysis (Tables 3 and 5).

As most patients in both groups were right handers, the differences in VLSM results between the LHD and RHD groups are likely to point to non-symmetrical physiological mechanisms underlying motor recovery in damage of the dominant vs. the non-dominant hemisphere. This conclusion is supported by the following facts: (a) the findings are based on relatively large samples of first-event LHD and RHD stroke patients, compared to previous VLSM studies of sensorimotor function post stroke [30,31]. Yet, it should be noted, that VLSM effects in small sample sizes of n = 30 and n = 60 can greatly overestimate as well as under-estimate effect sizes (based on analysis that used thousands of bootstrap samples from the same data set) [84]; (b) patients in both groups were recruited using the same strict inclusion criteria; (c) there were no significant differences between the two groups in possible confounding demographic and lesion variables (male/female proportion, age, motor dominance, stroke type, average lesion volume); (d) there were no significant differences between the two groups in the baseline FM and B&B scores; (e) in each anatomical region where ‘significant’ voxel clusters emerged in VLSM analysis in the RHD but not in the LHD group, the proportion of patients having a lesion (yes/no) did not differ between the groups (the only exception is the caudate nucleus, where in the chronic phase and in the Delta group proportionally more LHD than RHD patients had it involved); (f) among the anatomical structures where ‘significant’ voxel clusters emerged in the VLSM analysis, the only regions where the extent of damage differed between the groups were the posterior and retrolenticular parts of the internal capsule, which however were implicated in both groups (larger lesion extent in the subacute LHD group).

The above (a-f) facts suggest that the differences in lesion effects between LHD and RHD groups, shown here by VLSM, did not emerge due to a selection bias in the sample but may reflect a biological reality, probably related to hemispheric differences in motor control [85,86]. Left hemisphere dominance for skilled movement is attributed to anatomical and functional hemispheric asymmetries of the primary motor cortex and descending pathways as well as to asymmetry in premotor and association cortical areas [87]. Such differences are reflected in the gyral-sulcal architecture of the sensory-motor cortex [88], in the more extensive motor map in the hemisphere contralateral to the preferred hand [89], the more extensive connectivity of the left M1 with other parts of the brain [90], the higher excitability of the corticospinal system on the left [91], and the relationship between lateralization of the motor network and quality of performance of different motor tasks [92]. Motor cortex fMRI activation patterns following stroke have also been shown to exhibit hemispheric differences [32]. Thus, we propose that the paucity of ‘significant’ voxels in LHD relative to RHD VLSM (with no significant difference between the hemispheres neither in total nor in regional extent of damage) may stem from the fact that in the dominant left hemisphere, processing of sensory-motor data is done by a more widespread and more densely connected network [90], where damage to one component is more easily substituted by other network components (though, no such substitution is expected for large lesions affecting multiple network hubs). The more densely connected left motor network may bear some advantage for recovery processes, as hinted by the fact that delayed FM gain in the LHD Delta group was twice that of RHD Delta patients (9.76 ± 14.91 and 4.04 ± 9.69, respectively) despite equal average scores in the subacute and (LHD: 36.71 ± 21.19, RHD: 36.55 ± 22.53) and in the chronic phases (LHD: 44.31± 24.57, RHD: 45.79 ± 17.85), though the difference did not reach significance due to small sample size and large variance.

The difference in lesion effects between the LHD and RHD groups, revealed here in VLSM, holds only partial similarity to earlier studies, with variance possibly stemming from assessments being conducted at different times after stroke onset and selection of different measures to assess residual function (i.e., measures of basic motor impairment, like the FM used here, vs. measures of activity limitation and restricted participation, like the modified Rankin Scale, that are more likely to show amelioration gained by compensatory means) [11,12,93]. Irrespective of the assessment methods in use, repeated occurrence in research of LHD-RHD differences in recovery patterns points to inadequacy of the practice of pooling RHD and LHD lesion data together in studies assessing the impact of lesion location on motor recovery [30]. It should be taken into consideration, though, that the difference in lesion effects between the LHD and RHD groups found here were not based on analyses of all the affected voxels in the left and right hemispheres, as only voxels affected by at least 20% of the sample were analyzed (see threshold [marked by “T”] for inclusion in the VLSM analysis in Fig 1) and only ‘significant’ voxels forming clusters of at least 10 adjacent voxels were reported here.

### Limitations of the study

Assessment of lesion impact on task performance using the VLSM methodology is subject to different kinds of errors. Given the need to correct for multiple comparisons (as the basic anatomical unit in the analysis is a small volume of tissue, the ‘voxel’), false negative results are common, especially in case of small patient samples with large inter-personal variance, as in the chronic and delta groups of the current study. In addition, low-powered studies (due to small sample sizes) can also greatly overestimate as well as under-estimate effect sizes [84]. The results in the current study that did not pass the FDR correction for multiple comparisons are likely to point to a trend that may become significant with larger numbers of subjects, yet they may also exhibit type-1 errors. Correction for multiple comparisons by FDR, although used in past studies, is currently considered too liberal and is often substituted by permutation thresholding [94–96]. In the current study only the RHD group survived this correction in the VLSM analysis. The current study, and earlier VLSM studies aimed to assess the impact of damage to different brain structures on motor recovery, do not point to exactly the same voxels [11,12,30,31]. Beyond the above methodological issues, differences between studies in—initial severity of functional impairment; tools used to assess motor function; time point of behavioral testing; statistical analyses used; type, intensity and duration of rehabilitation treatment, and post-rehabilitation patients’ compliance with daily maintenance exercises–all these largely uncontrolled factors could affect the results of VLSM analyses [94,95,97]. In addition, the behavioral tests used here (especially the B&B) are likely to bear a different sensitivity to mild/moderate impairment of the dominant vs the non-dominant hand. Finally, as the first testing of patients in the current study was conducted in the subacute and not in the acute period, it is understood that structure-function relationships have already been modulated at this stage by practice-induced plasticity, as all these patients underwent at this stage intensive rehabilitation [22]. Comparing the effects of lesions on HUL functioning immediately after stroke onset vs. in the chronic phase would have probably better clarified lesion effects in their relation to recovery processes, i.e., the impact of damage to structures involved in motor control in the healthy brain vs. damage to structures taking place in adaptive remapping processes underlying motor recovery.

### Conclusions

The current study sheds new light on distinctions between the functional neuroanatomy underlying proximal and distal HUL motor control. It also points to differences between the functional neuroanatomy underlying motor recovery following left and right hemisphere damage. Finally, it corroborates earlier findings showing an effect of the time after stroke onset (subacute, chronic) on the results of VLSM analyses. Further studies are required for validation of trends pointed by the current results.

## Supporting information

S1 Table Individual demographic and clinical data from the subacute phase. *These patients were left handers; **These patients were ambidextrous; S—subacute; C—chronic; M—male; F—female; MCA—middle cerebral artery; ACA—anterior cerebral artery; PCA—posterior cerebral artery; BA—basilar artery; WS–water shadow; H—hemorrhagic stroke; I—ischemic stroke; I/H—ischemic with hemorrhagic transformation; TAO—time after stroke onset (months); FM—Fugl-Meyer (see Methods); NA–not applicable (several chronic patients were assessed by the FM for the first time in the chronic phase. Therefore, no data is available from their subacute phase); VFD—visual field defect (− = no VFD; −/e = extinction upon bilateral simultaneous stimulation but no VFD); Diagnosis of neglect was determined according to the Behavioral Inattention Test for neglect (43–44); Diagnosis of aphasia was determined according to the Israeli Loewenstein Aphasia Test (a comprehensive aphasia test battery for Hebrew-speaking patients; 45).
(DOC)

S2 Table VLSM results in RHD patients (n = 65) at the subacute phase using permutation correction. * FM A, FM B+C and FM T shown results passed permutation correction (corresponding in this analysis to a z score of 3.73, 3.68 and 3.65). // ALIC = anterior limb of internal capsule; EC = external capsule; SCR = superior corona radiata; SFO = superior fronto-occipital fasciculus; SLF = superior longitudinal fasciculus. Voxels`number in each behavior is listed from highest to lowest. For B&B—n = 38.
(DOCX)

S3 Table VLSM results in RHD patients (n = 65) at the chromic phase using permutation correction. * FM A, FM B+C, FM T and B&B shown results passed permutation correction (corresponding in this analysis to a z score of 4.16, 4.24, 4.12 and 3.87). // EC = external capsule; PCR, SCR = posterior, superior corona radiate; PLIC, RLIC = posterior, retrolenticular limb of internal capsule; SLF = superior longitudinal fasciculus. Voxels`number in each behavior is listed from highest to lowest. For B&B—n = 29.
(DOCX)

S1 Fig Individual lesion data. Each patient's lesion marked on arrays of 11 standard templates. Displays follow neurological conventions, i.e., right sided damage displayed on the left and left sided damage displayed on the right side. Only CT slices containing brain damage are shown. Green bar = Subacute group, Blue bar = Chronic group, Red bar = Delta group. In the case of a very small lesion, the MEDx system does not depict the lesion in the restricted set of standard templates used to present the structural damage. This happened in four patients: Patient number 2002 of the RHD group, lesion size 1.18cc, structure affected–corticospinal tract (CST) in its passage in the right ventral pons; Patient number 2013 of the RHD group, lesion size 1.21cc, structure affected–CST in its passage in the right ventral pons; Patient number 2016 of the RHD group, lesion size 0.35cc, structures affected CST in its passage in the right ventral pons and cerebral peduncle (CP); Patient number 2022 of the RHD group, lesion size 0.76cc, structure affected CST in its passage in the right ventral pons; Patient number 2044 of the RHD group, lesion size 0.28 cc, structures affected right middle CP; Patient number 1038 of the LHD group, lesion size 0.45, structures affected CST in its passage in the left ventral pons.
(TIF)
