# Supplementary material for: Lesion location impact on functional recovery of the hemiparetic upper limb
Source: PLoS One. 2019 Jul 19;14(7):e0219738. doi: 10.1371/journal.pone.0219738 (PMC6641167; doi:10.1371/journal.pone.0219738)
Supplement: S1 Table — *These patients were left handers; **These patients were ambidextrous; S—subacute; C—chronic; M—male; F—female; MCA—middle cerebral artery; ACA—anterior cerebral artery; PCA—posterior cerebral artery; BA—basilar artery; WS–water shadow; H—hemorrhagic stroke; I—ischemic stroke; I/H—ischemic with hemorrhagic transformation; TAO—time after stroke onset (months); FM—Fugl-Meyer (see Methods); NA–not applicable (several chronic patients were assessed by the FM for the first time in the chronic phase. Therefore, no data is available from their subacute phase); VFD—visual field defect (− = no VFD; −/e = extinction upon bilateral simultaneous stimulation but no VFD); Diagnosis of neglect was determined according to the Behavioral Inattention Test for neglect (43–44); Diagnosis of aphasia was determined according to the Israeli Loewenstein Aphasia Test (a comprehensive aphasia test battery for Hebrew-speaking patients; 45). (DOC) [file pone.0219738.s001.doc]

**Title: Individual demographic and clinical data from the subacute phase**

| Patient | Phase | Age/sex | Lesion side  and  Territory | Lesion  type | TAO  (months) | Lesion volume (cc) | FM A  (-/30) | FM B+C  (-/24) | FM T  (-/66) | FM Sensation  (-/12) | Visual  Field  Defect | Neglect | Aphasia |
| --- | --- | --- | --- | --- | --- | --- | --- | --- | --- | --- | --- | --- | --- |
| 1000 | C | 70/M | L-MCA | I | 33.25 | 111.61 | 21 | 14 | 43 | 6 | - | - | + |
| 1001 | S,C | 70/F | L-MCA | I | 1.74 | 34.34 | 29 | 23 | 62 | 11 | - | - | + |
| 1002 | S,C | 55/M | L-MCA | H | 2.39 | 20.73 | 11 | 10 | 27 | 0 | - | - | + |
| 1003 | S,C | 46/M | L-MCA | I | 1.18 | 15.2 | 29 | 23 | 64 | 12 | - | - | + |
| 1004 | S,C | 55/M | L-MCA | I | 1.08 | 35.6 | 9 | 6 | 15 | 12 | - | - | + |
| 1005 | C | 39/F | L-MCA | H | 52.20 | 40.57 | 21 | 16 | 46 | 8 | - | - | + |
| 1007 | S,C | 61/M | L-MCA | I | 2.33 | 1.42 | 4 | 6 | 14 | 12 | - | - | - |
| 1008 | S,C | 64/M | L-MCA | H | 1.11 | 75.28 | 18 | 17 | 39 | 9 | - | - | + |
| 1009 | S,C | 43/F | L-MCA | H | 1.61 | 45.54 | 11 | 6 | 21 | 6 | - | - | + |
| 1010 | S,C | 63/F | L-MCA | I | 2.92 | 74.31 | 27 | 11 | 46 | 12 | - | - | + |
| 1011 | S,C | 69/M | L-MCA | H | 1.48 | 69.21 | 0 | 0 | 4 | 8 | -/e | - | + |
| 1012 | S,C | 64/M | L-MCA | i | 1.90 | 5.59 | 26 | 23 | 59 | 12 | - | - | - |
| 1013 | S | 42/M | L-MCA | I | 0.89 | 2.19 | 29 | 16 | 53 | 10 | - | - | - |
| 1014 | S | 24/F | L-MCA | I | 1.02 | 26.04 | 5 | 0 | 9 | 12 | - | - | + |
| 1016 | S | 71/M | L-MCA | I>H | 1.70 | 15.42 | 22 | 19 | 44 | 10 | - | - | - |
| 1017 | S | 60/M | L-MCA | I | 3.05 | 40.4 | 17 | 21 | 47 | 12 | - | - | + |
| 1018 | S | 62/M | L-MCA | H | 1.93 | 9.08 | 0 | 0 | 0 | 12 | - | - | + |
| 1019 | S | 65/F | L-MCA | I | 1.02 | 1.65 | 15 | 9 | 32 | 12 | - | - | - |
| 1020 | S | 65/F | L-MCA | I | 1.38 | 53.66 | 17 | 17 | 41 | 5 | - | - | + |
| 1021 | S | 57/F | L-ACA | H | 1.51 | 22.19 | 2 | 7 | 13 | 12 | - | - | + |
| 1022 | S | 67/F | L-MCA | I | 0.79 | 17.16 | 29 | 23 | 60 | 11 | - | - | - |
| 1023 | S | 81/F | L-MCA | I | 1.15 | 12.53 | 28 | 23 | 58 | 12 | -/e | - | + |
| 1024 | S | 69/M | L-ACA/MCA WS | I | 1.44 | 47.14 | 16 | 6 | 29 | 11 | - | - | - |
| 1025 | S | 66/F | L-MCA | I | 1.84 | 5.18 | 12 | 13 | 30 | 12 | - | - | - |
| 1026 | S | 65/M | L-MCA | I | 2.00 | 5.05 | 19 | 15 | 42 | 12 | - | - | + |
| 1027 | S | 72/M | L-MCA | I | 1.28 | 50.24 | 28 | 20 | 56 | 12 | - | - | - |
| 1028 | S | 60/M | L-MCA | H | 1.44 | 57.88 | 30 | 24 | 66 | 10 | - | - | + |
| 1030* | S | 53/M | L-MCA | H | 0.82 | 22.23 | 2 | 0 | 6 | 1 | - | - | - |
| 1031 | S | 63/F | L-MCA | I | 0.56 | 6.8 | 24 | 18 | 48 | 8 | - | - | - |
| 1033* | S | 61/F | L-MCA | I | 0.82 | 25.71 | 9 | 0 | 13 | 12 | - | - | - |
| 1034 | S | 64/M | L-ACA | I | 0.72 | 33.6 | 17 | 9 | 45 | 12 | - | - | + |
| 1035 | S | 68/M | L-MCA | I | 1.21 | 12.68 | 2 | 0 | 2 | 4 | - | - | + |
| 1036 | S | 60/M | L-MCA | I | 0.46 | 1.16 | 23 | 22 | 52 | 5 | - | - | - |
| 1037 | S | 79/M | L-MCA | I | 0.69 | 1.15 | 1 | 0 | 5 | 12 | -/e | - | + |
| 1038 | S,C | 59/M | L-BA | H | 0.92 | 0.45 | 21 | 23 | 53 | NT | - | - | - |
| 1039 | S | 53/F | L-MCA | H | 1.64 | 3.12 | 23 | 21 | 50 | NT | - | - | - |
| 1040 | S | 57/M | L-MCA | I | 1.31 | 1.39 | 16 | 16 | 36 | NT | - | - | + |
| 1041 | S | 58/F | L-MCA | I | 0.95 | 1.96 | 12 | 0 | 16 | NT | - | - | - |
| 1042 | S,C | 65/M | L-MCA | H | 1.57 | 9.83 | 11 | 1 | 16 | NT | - | - | + |
| 1043 | S,C | 77/M | L-MCA | I | 1.02 | 18.54 | 28 | 24 | 62 | NT | - | + | + |
| 1044 | S | 67/M | L-MCA | I | 1.28 | 6.92 | 15 | 12 | 35 | NT | - | - | - |
| 1045 | S,C | 67/M | L-MCA | I | 0.56 | 9.57 | 27 | 24 | 62 | NT | - | - | + |
| 1046 | S | 64/M | L-PCA | I | 1.21 | 123.19 | 25 | 19 | 53 | NT | + | - | + |
| 1047 | S | 75/M | L-MCA | I | 0.79 | 52.12 | 28 | 22 | 62 | NT | - | - | + |
| 1048 | S,C | 26/M | L-MCA | I | 1.18 | 5.21 | 26 | 24 | 61 | NT | - | - | - |
| 1049 | C | 60/M | L-MCA | I | 17.11 | 4.02 | 9 | 0 | 11 | NT | - | - | + |
| 1050 | S | 32/F | L-MCA | I | 1.31 | 1.81 | 28 | 22 | 61 | NT | - | - | - |
| 1051 | S,C | 49/ | L-MCA | H | 0.92 | 7.13 | 11 | 0 | 19 | NT | - | - | + |
| 1052 | S | 64/M | L-MCA | I | 2.20 | 11.82 | 26 | 21 | 57 | NT | - | - | - |
| 1053 | S | 75/F | L-MCA | I | 1.74 | 3.54 | 27 | 19 | 56 | NT | - | - | + |
| 1054 | S | 75/M | L-MCA | I | 2.00 | 7.64 | NT | NT | 6 | NT | - | - | - |
| 1055 | S | 60/M | L-MCA | I | 2.23 | 6.14 | NT | NT | 5 | NT | - | - | - |
| 1056 | S | 69/M | L-MCA | I | 1.18 | 7.81 | 15 | 2 | 21 | 12 | - | - | - |
| 1057 | S | 54/F | L-MCA-PCA WS | I | 2.23 | 22.91 | 30 | 24 | 66 | NT | - | - | - |
| 1058 | S,C | 57/F | L-MCA | I | 1.64 | 7.27 | 20 | 16 | 46 | NT | - | - | + |
| 1059 | S,C | 46/M | L-MCA | I | 1.93 | 5.23 | 14 | 5 | 25 | NT | - | - | - |
| 1060 | S | 53/M | L-MCA | I | 2.39 | 4 | 5 | 2 | 14 | NT | - | - | - |
| 1061 | S,C | 46/ | L-MCA | H | 1.97 | 11.61 | 10 | 6 | 22 | NT | - | - | + |
| 1062 | S | 65/M | L-MCA | I | 1.15 | 5.86 | 30 | 24 | 66 | NT | - | - | - |
| 1063 | S,C | 66/M | L-MCA | H | 1.11 | 29.65 | 6 | 9 | 24 | 5 | - | - | + |
| 1064 | S,C | 57/M | L-MCA | H | 1.80 | 10.6 | 14 | 16 | 38 | 12 | - | - | - |
| 1065 | S,C | 67/M | L-MCA | H | 1.87 | 13.86 | 4 | 3 | 15 | 6 | - | + | + |
| 1066 | S | 33/M | L-MCA | I | 1.48 | 7.19 | 30 | 24 | 65 | 12 | - | - | - |
| 1067 | S | 60/M | L-MCA | I | 1.34 | 6.52 | 23 | 22 | 55 | 11 | - | - | - |
| 1069 | S,C | 63/M | L-MCA | H | 1.38 | 4.67 | 23 | 15 | 45 | 5 | - | - | - |
| 1070 | S,C | 60/M | L-MCA | I | 0.95 | 41.37 | 30 | 23 | 58 | 12 | - | - | + |
| 1071 | S,C | 59/M | L-MCA | I | 0.46 | 8.3 | 0 | 0 | 4 | 12 | - | - | + |
| 1072* | S | 69/M | L-MCA | I | 0.79 | 4.46 | 6 | 0 | 10 | 12 | - | - | - |
| 2000 | S,C | 50/F | R-MCA | I | 0.75 | 12.43 | 18 | 12 | 34 | 2 | - | - | - |
| 2001 | S,C | 64/M | R-MCA | H | 2.16 | 23.39 | 12 | 13 | 31 | 2 | -/e | - | - |
| 2002 | S,C | 63/F | R-BA | I | 1.41 | 1.18 | 12 | 3 | 21 | 12 | - | - | - |
| 2003 | S,C | 56/M | R-MCA | H | 1.05 | 1.19 | 22 | 16 | 42 | 0 | - | - | - |
| 2004 | S,C | 23/M | R-MCA | I | 0.95 | 0.98 | 30 | 24 | 66 | 12 | - | - | - |
| 2005 | S,C | 67/M | R-MCA | I>H | 2.33 | 93.16 | 0 | 2 | 4 | 0 | - | - | - |
| 2006 | C | 66/M | R-MCA | I | 31.02 | 92.41 | 30 | 24 | 64 | 12 | -/e | - | - |
| 2007* | S | 65/M | R-MCA | I | 2.66 | 34.34 | 0 | 0 | 4 | 10 | - | - | - |
| 2008 | S | 67/M | R-MCA | I | 1.31 | 10.89 | 0 | 0 | 4 | 12 | - | - | - |
| 2009 | S | 76/F | R-MCA | I | 2.69 | 3.76 | 18 | 17 | 43 | 12 | - | - | - |
| 2010 | S | 53/F | R-ACA | I | 2.20 | 41.93 | 0 | 13 | 17 | 8 | - | + | - |
| 2013 | S | 57/F | R-BA | H | 1.02 | 1.21 | 0 | 1 | 5 | 8 | - | - | - |
| 2014 | S | 71/M | R-MCA | H | 2.92 | 19.6 | 2 | 10 | 20 | 4 | - | - | - |
| 2015 | S | 60/M | R-MCA | I | 1.18 | 9.76 | 15 | 3 | 22 | 12 | - | - | - |
| 2016 | S | 79/F | R-BA | I | 0.89 | 0.35 | 28 | 24 | 60 | 10 | - | - | - |
| 2017 | S | 63/M | R-MCA | H | 2.00 | 96.64 | 19 | 17 | 46 | 12 | -/e | - | - |
| 2018 | S | 50/M | R-MCA | I | 0.56 | 5.42 | 22 | 23 | 49 | 12 | - | - | - |
| 2019 | S | 75/F | R-MCA | I | 2.39 | 43.54 | 7 | 7 | 18 | 8 | - | + | - |
| 2020 | S | 56/M | R-MCA | H | 2.59 | 56.13 | 0 | 0 | 4 | 8 | - | - | - |
| 2021 | S | 62/M | R-MCA | I | 0.85 | 2.23 | 19 | 14 | 41 | 12 | - | - | - |
| 2022 | S | 50/F | R-BA | I | 0.89 | 0.76 | 5 | 2 | 11 | 10 | - | - | - |
| 2023 | S | 38/F | R-MCA | I | 0.59 | 1.77 | 15 | 0 | 19 | 11 | - | - | - |
| 2024 | S | 69/M | R-MCA | I | 0.49 | 33.19 | 26 | 15 | 47 | 6 | - | - | - |
| 2025 | S | 44/M | R-MCA | I | 1.64 | 2.88 | 15 | 0 | 19 | 12 | - | - | - |
| 2026 | S | 46/F | R-MCA | I | 0.75 | 7.11 | 8 | 0 | 12 | 9 | - | - | - |
| 2027 | S | 67/M | R-MCA | I | 1.77 | 19.76 | 4 | 0 | 8 | 8 | - | - | - |
| 2028 | S | 60/M | R-MCA | I | 1.74 | 146.88 | 11 | 2 | 17 | 12 | - | - | - |
| 2031 | S | 56/M | R-MCA | H | 1.02 | 6.12 | 24 | 16 | 44 | 11 | - | - | - |
| 2033* | S | 69/F | R-MCA | I | 1.54 | 1.22 | 6 | 0 | 10 | 12 | - | - | - |
| 2034 | S | 57/F | R-MCA | I | 1.93 | 47.13 | 23 | 12 | 43 | NT | -/e | - | - |
| 2035 | S | 74/F | R-MCA | I | 1.93 | 12.22 | 24 | 20 | 52 | NT | -/e | - | - |
| 2036** | S,C | 70/F | R-MCA | I | 1.11 | 70.55 | 22 | 21 | 52 | NT | -/e | - | - |
| 2038 | S | 60/M | R-MCA | H | 1.67 | 31.1 | 7 | 8 | 23 | NT | - | - | - |
| 2039 | S | 58/F | R-MCA | H | 1.44 | 47.11 | 23 | 19 | 49 | NT | - | + | - |
| 2040 | S | 59/M | R-PCA | I | 2.92 | 24.96 | 20 | 19 | 45 | NT | + | + | - |
| 2041 | S,C | 54/M | R-MCA | H | 1.02 | 4.86 | 29 | 24 | 65 | 12 | - | - | - |
| 2042 | S | 76/F | R-MCA | H | 1.34 | 32.17 | 27 | 23 | 60 | NT | + | + | - |
| 2043 | S,C | 66/M | R-MCA | I | 1.34 | 26.16 | 23 | 20 | 50 | NT | - | - | - |
| 2044 | S,C | 66/M | R-MCA | I | 0.69 | 0.28 | 26 | 23 | 58 | NT | - | - | - |
| 2045 | S | 57/M | R-MCA | I | 0.85 | 1.91 | 25 | 23 | 56 | NT | - | - | - |
| 2046 | S,C | 62/M | R-MCA | H | 1.87 | 13.52 | 22 | 20 | 48 | NT | - | - | - |
| 2047 | S,C | 60/F | R-MCA | H | 1.38 | 17.86 | 28 | 22 | 62 | NT | - | + | - |
| 2048 | S | 65/M | R-MCA | I | 1.02 | 1.84 | 27 | 24 | 59 | NT | - | - | - |
| 2049 | S,C | 58/ | R-MCA | H | 0.89 | 1.3 | 26 | 21 | 56 | NT | -/e | - | - |
| 2050 | S | 77/M | R-MCA | H | 1.05 | 27.2 | 10 | 5 | 20 | 0 | - | + | - |
| 2051 | S,C | 65/M | R-MCA | I | 0.75 | 1.89 | 30 | 24 | 66 | NT | - | - | - |
| 2052 | S,C | 67/M | R-MCA | I | 1.38 | 44.79 | 27 | 17 | 51 | 12 | + | + | - |
| 2053 | S,C | 59/M | R-MCA | I | 0.72 | 0.99 | 30 | 24 | 66 | NT | - | - | - |
| 2054** | S | 65/M | R-MCA | I | 0.79 | 1.61 | 30 | 24 | 66 | NT | - | - | - |
| 2055 | S,C | 51/F | R-MCA | I | 1.21 | 11.87 | 28 | 18 | 54 | NT | - | - | - |
| 2056 | S | 76/F | R-MCA | I | 1.61 | 0.81 | 30 | 24 | 66 | NT | - | - | - |
| 2057 | S,C | 72/F | R-MCA | I | 0.69 | 1.36 | 30 | 24 | 66 | NT | - | - | - |
| 2058 | C | 56/M | R-MCA | H | 19.41 | 24.57 | 6 | 0 | 12 | 7 | - | - | - |
| 2059 | S | 43/M | R-MCA | I | 1.38 | 0.67 | 30 | 24 | 66 | NT | - | - | - |
| 2060 | S,C | 58/M | R-MCA | I | 0.89 | 0.78 | 30 | 24 | 66 | NT | - | - | - |
| 2062 | S | 66/M | R-MCA | I | 1.48 | 5.04 | 9 | 7 | 22 | 12 | - | - | - |
| 2063 | S | 61/M | R-MCA | I | 0.56 | 72.48 | 0 | 0 | 4 | 9 | - | + | - |
| 2064 | S | 62/M | R-MCA | I | 1.05 | 2.07 | 28 | 21 | 57 | 12 | - | - | - |
| 2065 | S,C | 65/M | R-MCA | I | 0.56 | 4.7 | 29 | 20 | 56 | 10 | - | - | - |
| 2066 | S | 50/F | R-MCA | I | 0.66 | 4.81 | 11 | 0 | 17 | 12 | - | - | - |
| 2067 | S,C | 67/F | R-MCA | I | 1.38 | 12.41 | 6 | 0 | 10 | 12 | - | - | - |
| 2068 | S | 60/M | R-MCA | I | 1.31 | 15.5 | 0 | 0 | 4 | 12 | - | + | - |
| 2069 | S | 52/M | R-MCA | I | 0.56 | 63.94 | 30 | 24 | 64 | 6 | - | - | - |
| 2070* | S,C | 55/M | R-MCA | I | 1.18 | 92.09 | 0 | 0 | 4 | 5 | - | + | - |
| 2071 | S,C | 69/M | R-MCA | I | 0.72 | 186.89 | 0 | 0 | 4 | 3 | - | + | - |
| 2072 | S,C | 66/M | R-MCA | I>H | 1.02 | 80.85 | 0 | 0 | 4 | 9 | -/e | + | - |
| 2073 | S | 57/M | R-MCA | H | 0.66 | 12.61 | 22 | 21 | 47 | 4 | -/e | - | - |
| 2074* | C | 40/M | R-MCA | H | 39.02 | 69.57 | 3 | 0 | 7 | 2 | - | - | - |
| 1074 | C | 66/M | L-MCA | I | 36.49 | 57.77 | 23 | 16 | 45 | 4 | - | - | + |
| 1075* | C | 65/M | L-MCA | I | 34.75 | 22.87 | 30 | 24 | 66 | 12 | - | - | - |
| 1076 | C | 55/M | L-MCA | I | 18.49 | 1.26 | 23 | 21 | 54 | 12 | - | - | - |
| 1077* | C | 56/F | L-ACA | I | 19.41 | 0.97 | 28 | 22 | 62 | 12 | - | - | - |
| 2075 | C | 65/M | R-MCA | I | 45.93 | 1.45 | 27 | 20 | 53 | 4 | - | - | - |
| 2076 | C | 67/M | R-MCA | H | 39.87 | 30.92 | 0 | 0 | 4 | 2 | -/e | - | - |
| 2077 | C | 69/M | R-MCA | I>H | 10.23 | 40.93 | 11 | 6 | 27 | NT | -/e | + | - |
| 2078 | C | 68/M | R-MCA | I | 28.95 | 2.44 | 28 | 23 | 62 | 12 | - | - | - |
| 2079 | C | 70/M | R-MCA | I | 31.84 | 20.92 | 28 | 23 | 59 | 12 | - | - | - |
| 1079 | C | 51/F | L-MCA | I | 14.00 | 3.35 | 12 | 14 | 30 | 12 | - | - | + |
| 1080 | C | 59/F | L-MCA | I | 14.16 | 5.42 | 25 | 21 | 52 | 7 | - | - | + |
