# Supplementary material for: Lesion location impact on functional recovery of the hemiparetic upper limb
Source: PLoS One. 2019 Jul 19;14(7):e0219738. doi: 10.1371/journal.pone.0219738 (PMC6641167; doi:10.1371/journal.pone.0219738)
Supplement: S2 Table — * FM A, FM B+C and FM T shown results passed permutation correction (corresponding in this analysis to a z score of 3.73, 3.68 and 3.65). // ALIC = anterior limb of internal capsule; EC = external capsule; SCR = superior corona radiata; SFO = superior fronto-occipital fasciculus; SLF = superior longitudinal fasciculus. Voxels`number in each behavior is listed from highest to lowest. For B&B—n = 38. (DOCX) [file pone.0219738.s002.docx]

**Title: VLSM results in RHD patients (n = 65) at the subacute phase using permutation correction**

| Test | Structure | Z-value | X | Y | Z | Voxels | % area |
| --- | --- | --- | --- | --- | --- | --- | --- |
| FM A* | Putamen | 4.52 | 26 | -2 | 6 | 110 | 10.34 |
|  | SCR | 5.04 | 30 | -10 | 28 | 105 | 11.41 |
|  | Insula | 4.84 | 38 | 0 | 10 | 85 | 4.80 |
|  | SLF | 4.89 | 34 | -4 | 22 | 77 | 9.33 |
|  | EC | 4.49 | 34 | -2 | 8 | 55 | 11.80 |
|  | ALIC | 4.62 | 18 | -2 | 18 | 25 | 6.14 |
|  | Caudate | 4.76 | 20 | -4 | 20 | 19 | 1.91 |
|  | SFO | 4.76 | 20 | -4 | 20 | 15 | 25.42 |
| FM B+C* | SCR | 4.35 | 28 | -12 | 28 | 20 | 2.17 |
|  | Putamen | 4.21 | 26 | -2 | 6 | 18 | 1.69 |
| FM T* | Putamen | 4.44 | 26 | -2 | 6 | 83 | 7.80 |
|  | SCR | 4.65 | 30 | -14 | 30 | 74 | 8.04 |
|  | Insula | 4.56 | 38 | 0 | 10 | 54 | 3.05 |
|  | EC | 4.21 | 34 | -2 | 8 | 37 | 7.94 |
|  | SLF | 4.51 | 34 | -2 | 22 | 34 | 4.12 |
|  | ALIC | 4.17 | 18 | -2 | 18 | 12 | 2.95 |
|  | Caudate | 4.29 | 20 | -4 | 20 | 11 | 1.11 |
| B&B | VLSM did not yield significant voxels that pass permutation correction | | | | | | |
