# Supplementary material for: Lesion location impact on functional recovery of the hemiparetic upper limb
Source: PLoS One. 2019 Jul 19;14(7):e0219738. doi: 10.1371/journal.pone.0219738 (PMC6641167; doi:10.1371/journal.pone.0219738)
Supplement: S3 Table — * FM A, FM B+C, FM T and B&B shown results passed permutation correction (corresponding in this analysis to a z score of 4.16, 4.24, 4.12 and 3.87). // EC = external capsule; PCR, SCR = posterior, superior corona radiate; PLIC, RLIC = posterior, retrolenticular limb of internal capsule; SLF = superior longitudinal fasciculus. Voxels`number in each behavior is listed from highest to lowest. For B&B—n = 29. (DOCX) [file pone.0219738.s003.docx]

**Title of table 5: VLSM results in RHD patients (n = 32) at the chronic using permutation correction**

| Test | Structure | Z-value | X | Y | Z | Voxels | % area |
| --- | --- | --- | --- | --- | --- | --- | --- |
| FM A* | SCR | 8.47 | 30 | -14 | 30 | 288 | 31.30 |
|  | SLF | 6.95 | 32 | -16 | 30 | 117 | 14.18 |
|  | Insula | 6.34 | 34 | -12 | 16 | 77 | 4.35 |
|  |  | 5.28 | 38 | 14 | -8 | 20 | 1.13 |
|  | EC | 5.71 | 32 | -14 | 16 | 67 | 14.38 |
|  |  | 5.28 | 28 | 16 | 8 | 10 | 2.15 |
|  | Putamen | 5.71 | 28 | -10 | 14 | 41 | 3.85 |
|  |  | 5.28 | 28 | 16 | 8 | 15 | 1.41 |
|  | PLIC | 6.26 | 26 | -12 | 18 | 39 | 7.78 |
|  | PCR | 6.59 | 26 | -22 | 26 | 29 | 6.42 |
|  | RLIC | 5.35 | 36 | -26 | 2 | 21 | 6.65 |
|  |  | 5.27 | 28 | -24 | 14 | 15 | 4.75 |
| FM B+C* | SCR | 7.73 | 26 | -16 | 24 | 282 | 30.65 |
|  | SLF | 6.76 | 32 | -16 | 30 | 125 | 15.15 |
|  | Insula | 6.43 | 34 | -12 | 16 | 91 | 5.14 |
|  |  | 5.49 | 38 | 14 | -8 | 20 | 1.13 |
|  | EC | 5.62 | 32 | -14 | 16 | 80 | 17.17 |
|  | Putamen | 5.56 | 32 | -6 | 10 | 53 | 4.98 |
|  | PCR | 6.11 | 26 | -24 | 24 | 33 | 7.30 |
|  | PLIC | 5.77 | 26 | -12 | 18 | 24 | 4.79 |
|  | RLIC | 5.07 | 28 | -24 | 14 | 11 | 3.48 |
| FM T* | SCR | 8.32 | 30 | -14 | 30 | 305 | 33.15 |
|  | SLF | 7.19 | 32 | -16 | 30 | 147 | 17.82 |
|  | Insula | 6.41 | 34 | -12 | 16 | 87 | 4.92 |
|  |  | 5.21 | 38 | 14 | -8 | 22 | 1.24 |
|  | EC | 5.72 | 32 | -14 | 16 | 69 | 14.81 |
|  |  | 5.16 | 28 | 16 | 8 | 12 | 2.58 |
|  | Putamen | 5.43 | 28 | -10 | 14 | 41 | 3.85 |
|  |  | 5.16 | 28 | 16 | 8 | 17 | 1.60 |
|  | PLIC | 5.94 | 26 | -12 | 18 | 38 | 7.58 |
|  | PCR | 6.51 | 26 | -22 | 26 | 35 | 7.74 |
|  | RLIC | 5.35 | 36 | -26 | 2 | 21 | 6.65 |
|  |  | 5.27 | 28 | -24 | 14 | 15 | 4.75 |
| B&B* | SCR | 5.96 | 28 | -8 | 20 | 200 | 21.74 |
|  | EC | 5.68 | 32 | -14 | 16 | 83 | 17.81 |
|  | Insula | 5.68 | 34 | -12 | 16 | 69 | 3.90 |
|  | SLF | 5.52 | 32 | -16 | 30 | 55 | 6.67 |
|  | Putamen | 5.96 | 28 | -10 | 14 | 46 | 4.32 |
|  | PLIC | 5.96 | 28 | -12 | 14 | 36 | 7.19 |
|  | PCR | 4.90 | 26 | -22 | 26 | 15 | 3.32 |
