# Supplementary figures and images for: Lesion location impact on functional recovery of the hemiparetic upper limb
Source: PLoS One. 2019 Jul 19;14(7):e0219738. doi: 10.1371/journal.pone.0219738 (PMC6641167; doi:10.1371/journal.pone.0219738)

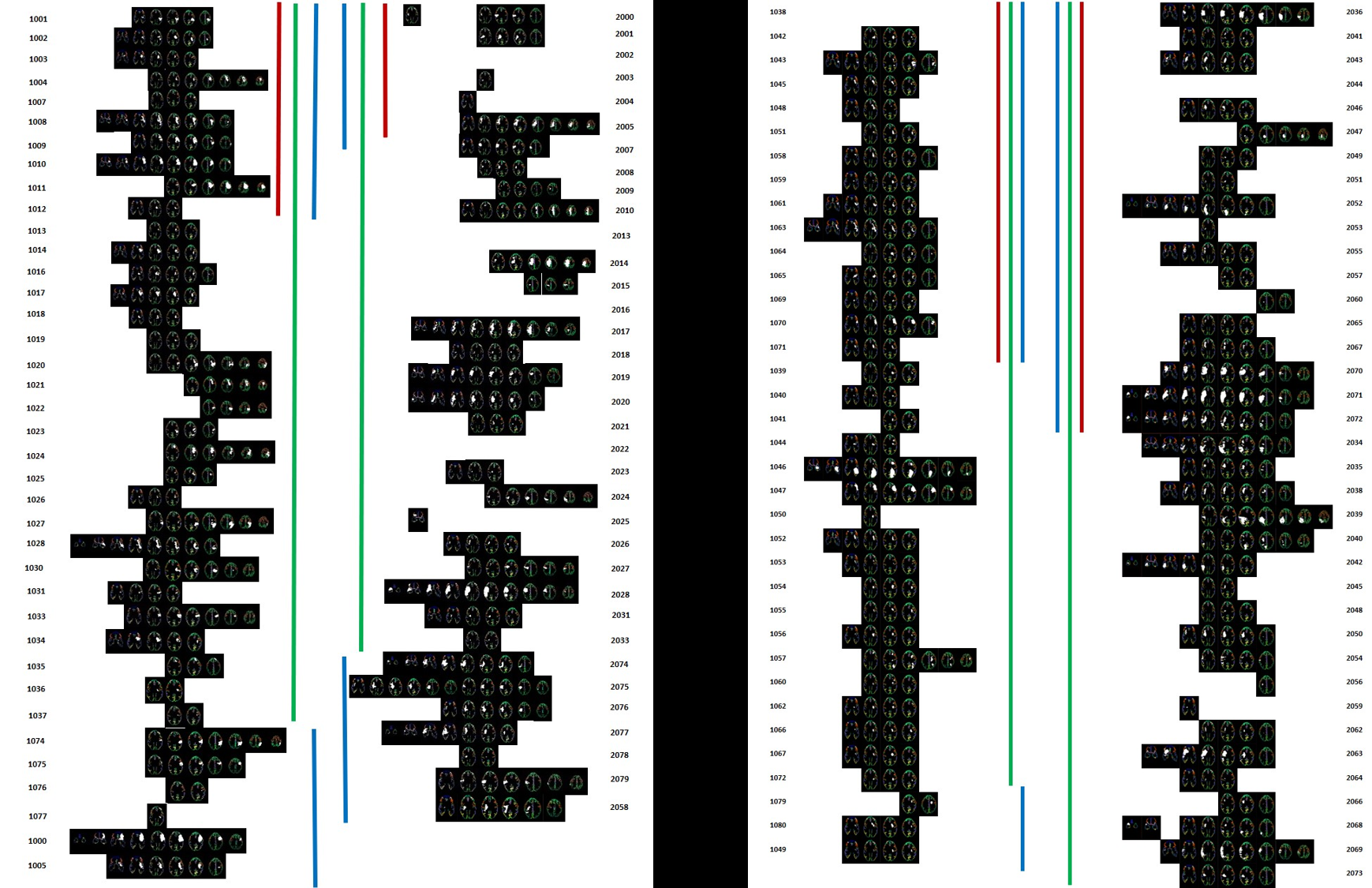

Supplement: S1 Fig — Each patient's lesion marked on arrays of 11 standard templates. Displays follow neurological conventions, i.e., right sided damage displayed on the left and left sided damage displayed on the right side. Only CT slices containing brain damage are shown. Green bar = Subacute group, Blue bar = Chronic group, Red bar = Delta group. In the case of a very small lesion, the MEDx system does not depict the lesion in the restricted set of standard templates used to present the structural damage. This happened in four patients: Patient number 2002 of the RHD group, lesion size 1.18cc, structure affected–corticospinal tract (CST) in its passage in the right ventral pons; Patient number 2013 of the RHD group, lesion size 1.21cc, structure affected–CST in its passage in the right ventral pons; Patient number 2016 of the RHD group, lesion size 0.35cc, structures affected CST in its passage in the right ventral pons and cerebral peduncle (CP); Patient number 2022 of the RHD group, lesion size 0.76cc, structure affected CST in its passage in the right ventral pons; Patient number 2044 of the RHD group, lesion size 0.28 cc, structures affected right middle CP; Patient number 1038 of the LHD group, lesion size 0.45, structures affected CST in its passage in the left ventral pons. (TIF) [file pone.0219738.s004.tif]
